# Concurrent Etching and Exchange Reactions During the Partial Replacement of Cu^+^ with Ni^2+^ in Copper Sulfide Nanorods

**DOI:** 10.1021/acs.inorgchem.6c03606

**Published:** 2026-09-09

**Authors:** Chul-Hyun Jeong, Raymond E. Schaak

**Affiliations:** † Department of Chemistry, The Pennsylvania State University, University Park, Pennsylvania 16802, United States; ‡ Department of Chemical Engineering, The Pennsylvania State University, University Park, Pennsylvania 16802, United States; § Materials Research Institute, The Pennsylvania State University, University Park, PA 16802, Unites States

## Abstract

Partially exchanging a fraction of the Cu^+^ cations in copper sulfide nanoparticles with other cations can produce heterostructured derivatives that incorporate multiple metal sulfides. Cation exchange reactions can compete with etching processes, where the exchanging cation facilitates dissolution of any remaining copper sulfide. Here, we show that during partial Ni^2+^ exchange of Cu_1.8_S nanorods to form Cu_1.8_S–Ni_*x*_S_*y*_ heterostructures, etching instead occurs in the Ni_*x*_S_*y*_ region that is undergoing exchange, rather than in Cu_1.8_S. Experiments that isolated individual exchange variables identified competing cation extraction, sulfur abstraction, and cation reincorporation processes that are conceptually related to reactions implicated in the hydrometallurgical processing of nickel–copper converter matte. These insights help to rationalize the unusual etching behavior observed during partial Ni^2+^ exchange of Cu_1.8_S.

## Graphical abstract

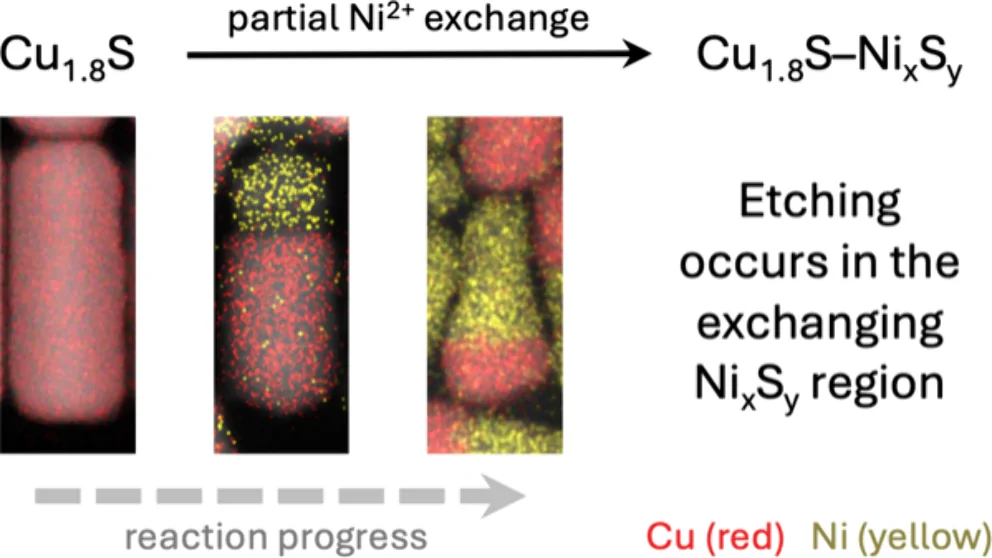

Partial cation exchange reactions provide a versatile synthetic platform for generating heterostructured nanoparticles that contain two or more compositionally distinct regions, leading to tailored properties that can be difficult to achieve through direct synthesis.^1,2^ Nanoparticles of roxbyite copper sulfide (Cu_1.8_S), which contain Cu^+^ cations, are useful cation exchange precursors. The Cu^+^ cations of Cu_1.8_S interact with trioctylphosphine (TOP) ligands through favorable hard-soft acid–base interactions, which drive exchange by solubilizing the Cu^+^ cations and creating transient cation vacancy sites that can then be occupied by harder cations that enter from solution.^2,3^ Substoichiometric and incomplete exchange results in heterometal sulfide nanoparticles, connected through interfaces.^4^

In this cation exchange process, benzyl ether, 1-octadecene, and oleylamine serve as solvents that also assist in stabilizing the nanoparticle surfaces.^4^ However, etching can accompany and compete with cation exchange under the same reaction conditions, though it is often overlooked when exchange chemistry dominates. For instance, during Ga^3+^ exchange using GaCl_3_, copper sulfide is selectively etched under certain reaction conditions. Such etching behavior has been observed in *M*_*x*_S_*y*_–Cu_1.8_S nanoparticles (*M* = Zn, Cu_0.5_In_0.5_, Cd) and in reactions employing triphenylphosphite and diphenylphosphine.^5,6^ TOP can selectively etch copper sulfide regions in ZnS–Cu_2‑x_S heterostructures through sulfur abstraction under oxidizing conditions.^7^ Etching can damage nanoparticle morphology, but controlled etching can produce sophisticated morphologies. Understanding nanoparticle etching chemistry is therefore important for both preventing unwanted degradation and designing intricate nanostructures.^8^

We observed, unexpectedly, that when Ni^2+^ was included in sequential partial exchange processes on Cu_1.8_S nanorods, the product morphologies were shortened or degraded,^9^ while analogous reactions without Ni^2+^ preserved the original morphology.^10^ Etching processes that compete with exchange typically involve degradation of the Cu_1.8_S regions.^5–7^ However, specific to the case of Ni^2+^, it is the exchanged nickel sulfide regions that are simultaneously degraded, rather than the Cu_1.8_S regions. To identify the factors responsible for this unusual behavior, we designed and analyzed a series of experiments that isolated individual exchange variables and enabled systematic composition-dependent morphological analysis.

Starting with roxbyite Cu_1.8_S nanorods (Figures 1a, S1), we performed single-step partial exchanges using Ni^2+^ alone (1/3), Co^2+^ alone (1/3), or Ni^2+^ and Co^2+^ simultaneously (1/6 each); parentheses indicate the mole fractions relative to Cu^+^ in the starting Cu_1.8_S nanorods. We used scanning transmission electron microscopy (STEM) to compare high-angle annular dark field (HAADF) images with energy dispersive X-ray spectroscopy (EDS) element maps. Exchange reactions involving Ni^2+^ produced visible nanorod degradation, whereas exchange with only Co^2+^ preserved the nanorod morphology, despite otherwise identical conditions (Figures 1, S2–S4). Additionally, after partial Ni^2+^ exchange, the nanoparticles contained predominantly nickel sulfide regions that were tapered, with only small regions of residual copper sulfide (Figure 1 **,** Table S1). In contrast, large Cu_1.8_S regions remained after partial Co^2+^ exchange, with nanorod dimensions approaching the original Cu_1.8_S precursors (Figure 1). This result suggests that during partial Ni^2+^ exchange, the nickel sulfide regions undergo etching that occurs concomitantly with exchange, such that Ni^2+^ replaces Cu^+^ on a similar time scale as the nickel sulfide is etched. Partial simultaneous Ni^2+^/Co^2+^ exchange displayed a combination of features from both single-cation reactions. The cobalt-containing tip regions, which likely form nickel–cobalt sulfide solid solutions,^11^ retained their unetched nanorod-tip shape, whereas the Cu_1.8_S regions showed only slight degradation, likely due to surface-level transient nickel sulfides during exchange (discussed below). The different partial exchange behaviors of Ni^2+^ and Co^2+^ are unlikely due to structural factors, as both generate closely related *M*_9_S_8_-type sulfides.^11^

**Figure 1:**
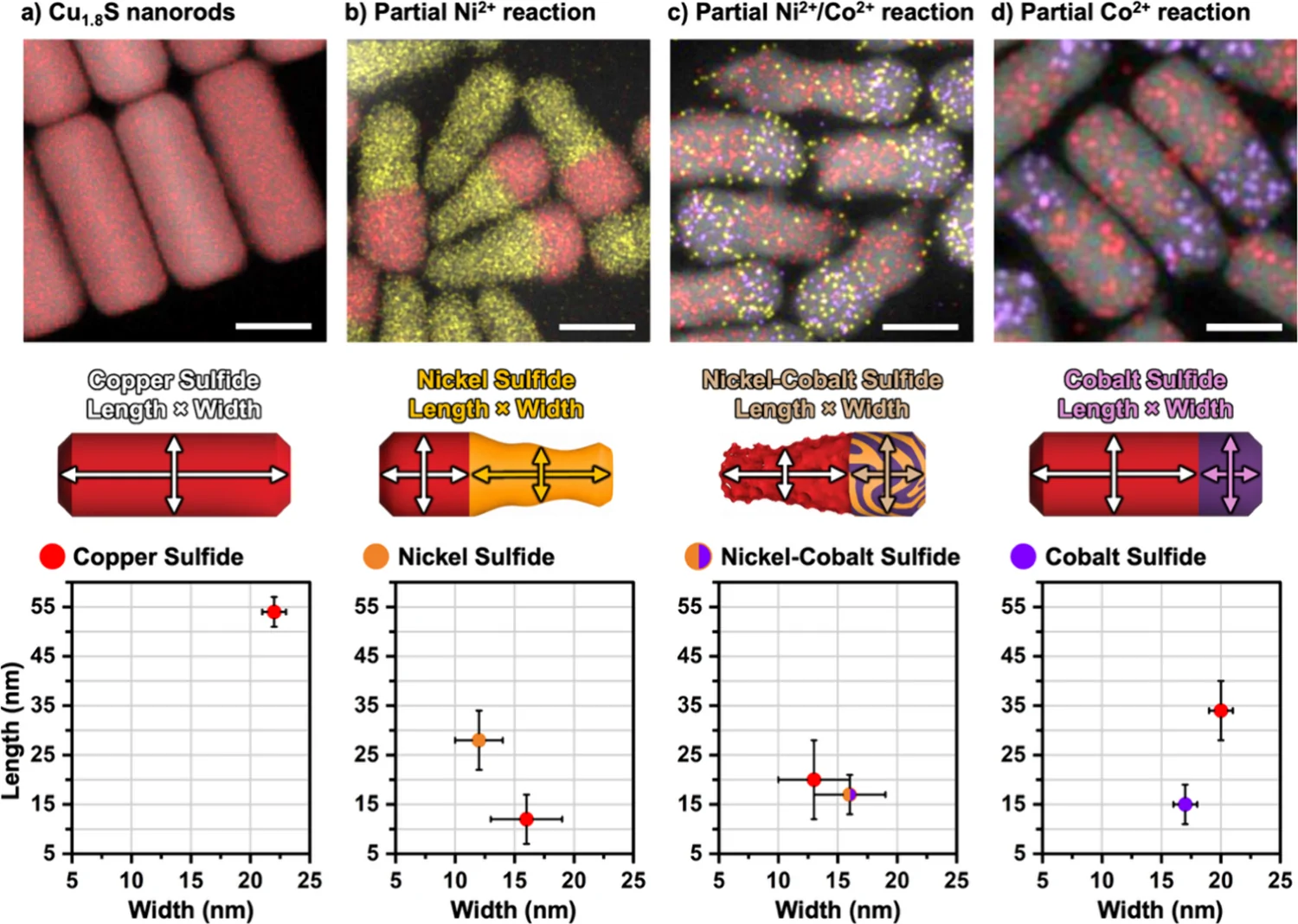
STEM-EDS element maps [CuKα (red), CoKα (purple), NiKα (yellow)] overlaid on HAADF-STEM images for (a) Cu_1.8_S nanorods and the products of (b) partial Ni^2+^, (c) partial Ni^2+^/Co^2+^, and (d) partial Co^2+^ reactions. The schematics define the length and width measurements, which are summarized in the plots. Scale bars are 20 nm.

To examine how nanoparticle etching evolves, we collected aliquots at 3, 17, and 50 min during 90 min partial exchange reactions with Ni^2+^ alone and with a Ni^2+^/Co^2+^ mixture (Figures 2, S5–S6). At 3 min, the nanorods exhibited largely intact morphologies, similar to those observed after Co^2+^ exchange. However, a darker-contrast and slightly narrowed nickel sulfide region appears near the interface, likely due to the onset of etching, and this feature became more prominent in later aliquots. Exchange and etching both emerge rapidly, making these processes difficult to fully deconvolute. By 17 min, the nickel sulfide regions directly interfaced with copper sulfide appeared narrower than the nickel sulfide regions farther away from the interface and toward the nanorod tips. Corresponding HAADF-STEM images showed slightly darker contrast in these interfacial nickel sulfide regions (Figures 2a, S5). This observation is consistent with the presence of less material in these regions, suggesting that nickel sulfide interfaced with copper sulfide may be less stable than nickel sulfide farther from the interface, making it a potential initiation point for etching. By 50 min, significant morphological degradation was observed. This observation suggests that as the exchange reaction progresses, etching becomes more widespread. STEM-EDS analysis indicated that the Ni:S ratio increased from 0.9:1 at 3 min to 1.2:1 at 90 min (Table S2), which is consistent with an etching process involving sulfur abstraction from the nickel sulfide regions. (Aliquots yielded insufficient quantities for XRD, so we refer to them as Ni_*x*_S_*y*_). Aliquots from simultaneous Ni^2+^/Co^2+^ exchange reactions showed a relatively intact morphology at 3 min but gradually exhibited etching at 17 and 50 min (Figure S6).

**Figure 2:**
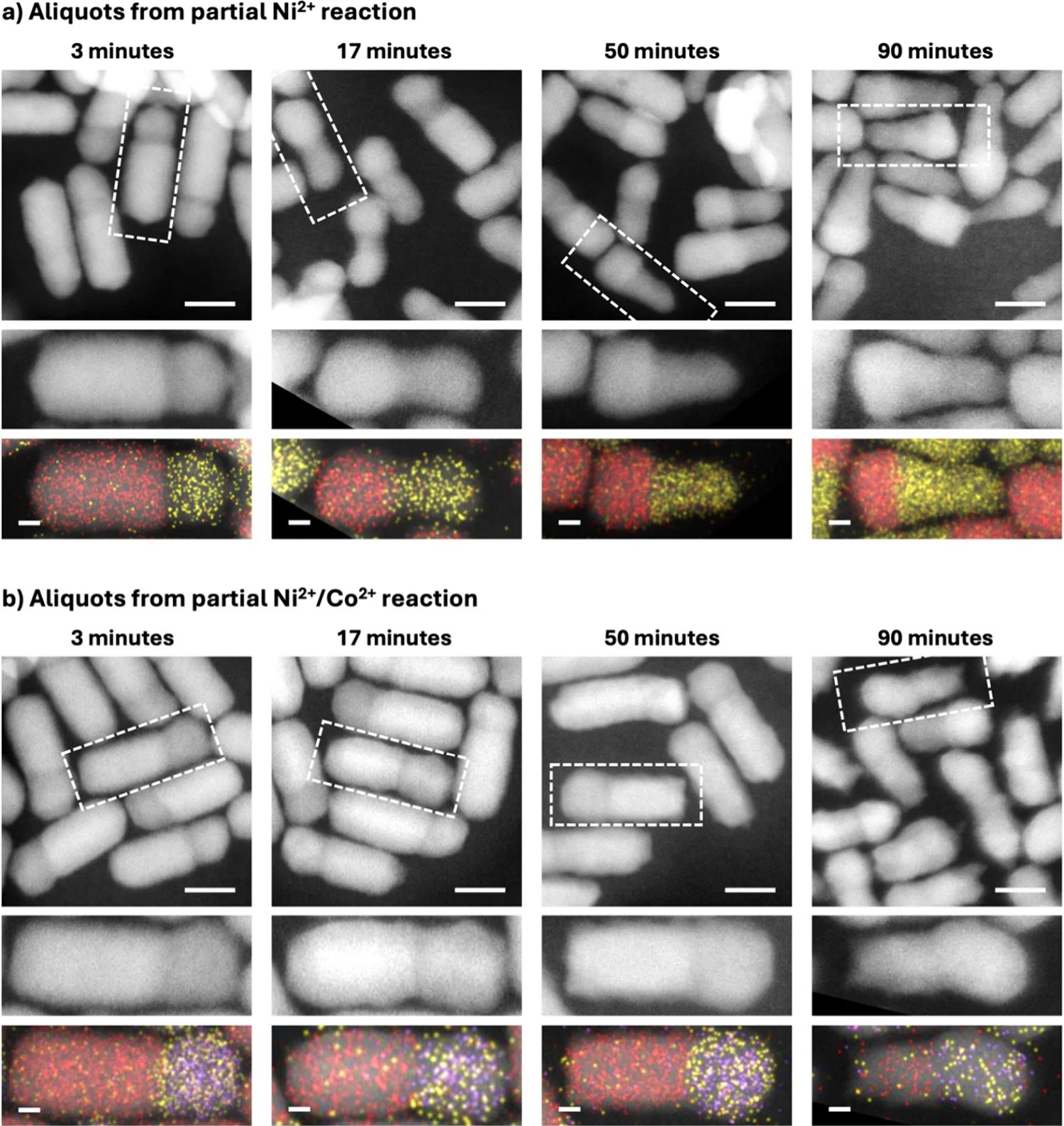
HAADF-STEM images and corresponding STEM-EDS element maps [CuKα (red), CoKα (purple), NiKα (yellow)] for aliquots collected during (a) partial Ni^2+^ and (b) partial Ni^2+^/Co^2+^ reactions. White dashed boxes highlight the enlarged particles shown below. HAADF-STEM and STEM-EDS scale bars are 20 and 5 nm, respectively.

Having established that etching occurs during partial Ni^2+^ exchange, we investigated the potential role of ligands. Oleylamine can extract Ni^2+^ from nickel sulfide nanoparticles and drive Cu^+^ back-exchange reactions.^12^ Literature studies further suggest that chloride anions, which are present in the NiCl_2_ reagent, could potentially facilitate etching of metal sulfides.^13,14^ We therefore designed experiments to probe how oleylamine and Cl^–^ contribute to etching, while maintaining TOP as the driving force for cation exchange (Figures 3a-**f**, Tables S3–S4).

**Figure 3:**
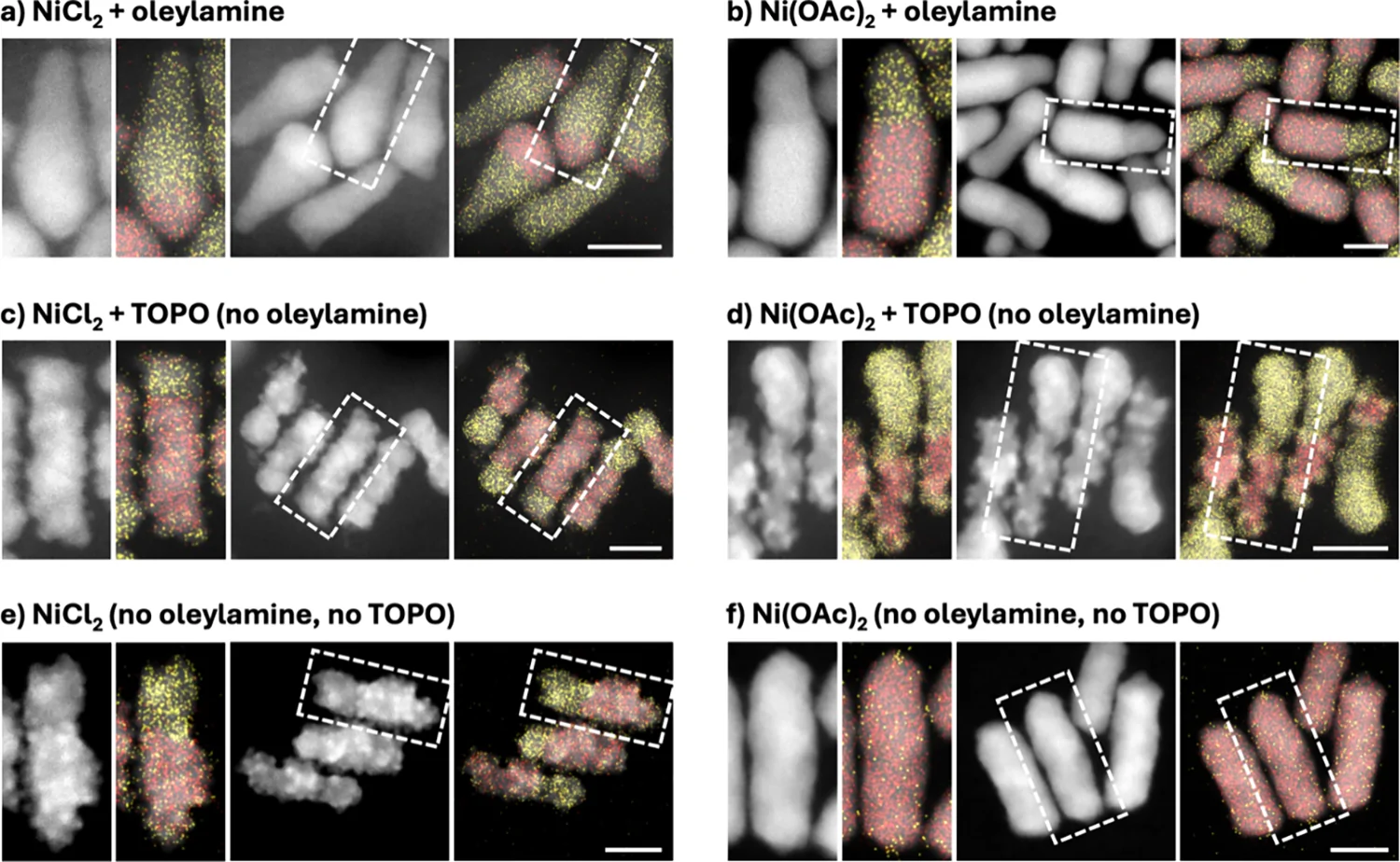
(a-f) HAADF-STEM images and overlaid STEM-EDS element maps [CuKα (red), NiKα (yellow)] of products obtained using the indicated reaction variables. White dashed boxes highlight the enlarged particles. Scale bars are 20 nm.

First, we tested Ni^2+^ exchange reactions without oleylamine but in the presence of Cl^–^ (Figures 3c, 3 **e**, S7–S8). Two reactions had Cl^–^ present; one used trioctylphosphine oxide (TOPO) instead of oleylamine and another used neither oleylamine nor TOPO. Both yielded similar morphologies and cation-exchanged regions. The nanorod tips exchanged to form distinct nickel sulfide regions, while the nanorod surfaces showed evidence of small nickel sulfide regions and slightly degraded copper sulfide regions, resulting in granular surfaces that may form through concurrent surface-level transient nickel sulfide formation and degradation during cation exchange, similar to the partial Ni^2+^/Co^2+^ reaction product in Figure 1c. This observation suggests that Ni^2+^ exchange reactions without oleylamine produce different morphological features, but concurrent cation exchange and etching processes cannot be circumvented simply by removing oleylamine.

Second, we replaced NiCl_2_ with nickel acetate, Ni­(OAc)_2_, to exclude Cl^–^ from the system. To directly compare these results with those of the NiCl_2_-based exchange, we conducted three parallel experiments with Ni­(OAc)_2_: one with oleylamine, one with TOPO, and one with neither oleylamine nor TOPO (Figures 3b, 3 **d**, 3 **f**, S9–S11). Ni­(OAc)_2_ with oleylamine generated products similar to the NiCl_2_-based reactions, though the effects were less pronounced (Figures 3a, 3 **b**, S2, S9). Ni^2+^ exchange using Ni­(OAc)_2_ with TOPO but without oleylamine yielded features very similar to when NiCl_2_ and TOPO were used. The nanorod tips exchanged to form nickel sulfide and the overall nanorod shape adopted a granular surface with small nickel sulfide deposits (Figures 3d, S10). However, Ni^2+^ exchange using Ni­(OAc)_2_ with neither oleylamine nor TOPO produced different results from the NiCl_2_-based reactions (Figures 3e, 3 **f**, S8, S11). This difference likely reflects the poor solubility of Ni­(OAc)_2_ in TOP under these conditions. Although NiCl_2_ was also initially present as an undissolved powder in the cation stock solution (Table S3), it is known to dissolve upon exposure to TOP.^15^ In contrast, the poor solubility of Ni­(OAc)_2_ appears to limit exchange, although mild surface degradation was still apparent (Figures 3f, S12). A control experiment using TOP but without added metal salt (Figure S13) showed that the nanorod surfaces remained unchanged, suggesting that even trace amounts of dissolved Ni­(OAc)_2_ can promote minor surface degradation. These ligand-variation experiments indicate that simply removing Cl^–^ or altering the presence of oleylamine is insufficient to suppress etching. Instead, the Ni^2+^ exchange reaction itself, which ultimately forms the nickel sulfide–copper sulfide heterostructure, may be the key factor that facilitates etching.

To gain further insight, we turned to related examples in the industrial hydrometallurgical processing of nickel–copper converter matte, where nickel and copper are extracted from heterogeneously mixed sulfide systems, including djurleite (Cu_1.94_S) and heazlewoodite (Ni_3_S_2_), through chemical leaching in acidic media.^16^ As leaching proceeds, continuous cation removal from the sulfides produces progressively more sulfur-rich phases such as covellite (CuS) and polydymite (Ni_3_S_4_) before complete dissolution.^16,17^ Because converter matte is a heterogeneous mixture of copper and nickel sulfides, the chemical leaching process for each phase is not independent but rather coupled through interfacial interactions,^17^ which has a structural analogy to our heterostructured nickel–copper sulfide nanorods. During this hydrometallurgical process, more oxidized sulfide phases can facilitate leaching of less oxidized sulfide phases, resulting in a sequential and iterative leaching process between different phases.^17,18^

The converter-matte comparison is intended as a conceptual analogy where interfaced copper sulfide and nickel sulfide domains can influence one another’s reactivity, rather than as a direct mechanistic equivalence. Our reaction generates interfaced copper sulfide–nickel sulfide heterostructures in a solution containing ligands capable of extracting both Cu and Ni cations; TOP extracts Cu^+^ and sulfur from copper and other metal sulfides,^3,7,19–21^ while oleylamine extracts Ni^2+^ from nickel sulfides.^12^ In addition, TOP and TOPO can interact with the Ni^2+^ in nickel sulfide as binding ligands.^22,23^ We therefore propose that the copper sulfide–nickel sulfide heterointerface provides a pathway through which the two sulfide domains become interdependent and destabilize each other, although the specific chemical steps are expected to differ from those involved in converter-matte leaching. The copper sulfide near the interface may facilitate a transient conversion of Ni_*x*_S_*y*_ toward more sulfur-rich nickel sulfide phases. However, this tendency would compete directly with the opposing effect of TOP, which favors sulfur abstraction and therefore a shift toward more metal-rich phases. We speculate that the coexistence of these opposing chemical driving forces promotes destabilization and etching of the nickel sulfide regions, with the process likely initiated near the interface, consistent with the darker and narrower nickel sulfide regions observed in Figure 2a.

This etching process, which itself involves competitive reactions, competes with cation exchange that can occur under the same conditions. Although nickel sulfide dissolution becomes more widespread throughout the reaction while preserving the heterointerface, the Ni^2+^ cations extracted by oleylamine can be reincorporated into the copper sulfide through cation exchange. This conjecture is consistent with the over-exchanged and tapered nanorods in Figure 2a, reflecting their formation through a coupled exchange-etching pathway rather than through simple cation exchange alone.

Experiments at different temperatures, designed to identify the nickel sulfide phase obtained through complete exchange, provide further support for these competing processes. Full Co^2+^ exchange at both 100 and 140 °C yielded pentlandite (Co_9_S_8_) nanorods, but for full Ni^2+^ exchange, godlevskite Ni_9_S_8_ formed at 100 °C while 140 °C produced mostly nickel-rich heazlewoodite (Ni_3_S_2_) (Figures S14–S15). Significant heazlewoodite peaks were also observed after complete Ni^2+^ exchange at 180 °C (Figure S14). The observation of Ni_3_S_2_ indicates that this nickel-rich phase is accessible during cation exchange and suggests that the proposed competing reaction pathway, in which cation extraction, sulfur abstraction, and Ni^2+^ reincorporation occur *en route* to dissolution, is plausible.

The concurrent etching and exchange behavior of substoichiometric reactions of NiCl_2_ with Cu_1.8_S nanorods can be rationalized by competing cation extraction, sulfur abstraction, and cation reincorporation processes, although structural disorder, strain, or defects introduced during exchange may also contribute. Removing oleylamine or/and Cl^–^, which are two key components of the reaction, does not suppress etching during partial Ni^2+^ exchange. This etching behavior differs from that of other cations,^5,6^ suggesting composition-dependent pathways. Coupled etching and exchange provides a potential design tool for precisely reshaping morphology and decreasing feature size directly during cation exchange, which could provide an entryway for installing quantum-confined components within larger semiconductor nanorod heterostructures.

## Supplementary Material



## Notes

Experimental details, Tables S1 through S4, and Figures S1 through S15, which include additional XRD, HAADF-STEM, STEM-EDS, and EDS data (PDF)

## References

[1] Saruyama M., Sato R., Teranishi T. (2021). Transformations of Ionic Nanocrystals
via Full and Partial Ion Exchange Reactions. Acc. Chem. Res..

[2] Schaak R. E., Steimle B. C., Fenton J. L. (2020). Made-to-Order Heterostructured
Nanoparticle
Libraries. Acc. Chem. Res..

[3] De
Trizio L., Manna L. (2016). Forging Colloidal Nanostructures
via Cation Exchange Reactions. Chem. Rev..

[4] Steimle B. C., Fagan A. M., Butterfield A. G., Lord R. W., McCormick C. R., Di Domizio G. A., Schaak R. E. (2020). Experimental Insights into Partial
Cation Exchange Reactions for Synthesizing Heterostructured Metal
Sulfide Nanocrystals. Chem. Mater..

[5] Hinterding S. O. M., Berends A. C., Kurttepeli M., Moret M.-E., Meeldijk J. D., Bals S., van der Stam W., de Mello Donega C. (2019). Tailoring
Cu^+^ for Ga^3+^ Cation Exchange in Cu_2–x_S and CuInS_2_ Nanocrystals by Controlling the Ga Precursor
Chemistry. ACS Nano.

[6] Veglak J. M., Benson N. A., Schaak R. E. (2025). Competitive
Cation Exchange and Etching
Processes in Metal Sulfide Nanoparticles from Anion/Ligand Cooperativity
and Exogenous Cations. J. Am. Chem. Soc..

[7] Nelson A., Ha D.-H., Robinson R. D. (2016). Selective Etching of Copper Sulfide
Nanoparticles and Heterostructures through Sulfur Abstraction: Phase
Transformations and Optical Properties. Chem.
Mater..

[8] Saleem F., Liu G., Liu G., Chen B., Yun Q., Ge Y., Zhang A., Wang X., Zhou X., Wang G. (2024). Crystal-Phase-Selective Etching of Heterophase Au Nanostructures. Small Methods.

[9] McCormick C. R., Katzbaer R. R., Steimle B. C., Schaak R. E. (2023). Combinatorial cation
exchange for the discovery and rational synthesis of heterostructured
nanorods. Nature Synthesis.

[10] Steimle B. C., Fenton J. L., Schaak R. E. (2020). Rational
construction of a scalable
heterostructured nanorod megalibrary. Science.

[11] Jeong C.-H., McCormick C. R., Schaak R. E. (2025). Solid Solution Formation from Sequential
Interfacial Reactions during Nanoparticle Cation Exchange. Chem. Mater..

[12] Suriyawansa D. N., McCormick C. R., Young H. L., O’Boyle S. K., Schaak R. E. (2025). Structure-Shifting
Intermediates During Nanoparticle
Cation Exchange for the Retrosynthetic Construction of Intraparticle
Heterophase Homojunctions. ACS Nano.

[13] Senanayake G. (2009). A review of
chloride assisted copper sulfide leaching by oxygenated sulfuric acid
and mechanistic considerations. Hydrometallurgy.

[14] Han J. H., Park M., Lee J.-u., Choi C., Park J. B., Lim Y., Kim G., Jung J., Lungerich D., Jun C.-H. (2025). Molecular
Drillers for 2 nm Resolution Nanochannel
Perforation of 2D Nanoplates. J. Am. Chem. Soc..

[15] Zaza L., Boulanger C., Kumar K., Agrachev M., Bornet A., Leemans J., Jeschke G., Buonsanti R. (2025). Ligand Electronic
Properties Dictate Composition of Ni-Based Nanocrystals. J. Am. Chem. Soc..

[16] Rademan J. A. M., Lorenzen L., van Deventer J. S. J. (1999). The
leaching characteristics of Ni–Cu
matte in the acid–oxygen pressure leach process at Impala Platinum. Hydrometallurgy.

[17] Provis J. L., van Deventer J. S. J., Rademan J. A. M., Lorenzen L. (2003). A kinetic model for
the acid-oxygen pressure leaching of Ni–Cu matte. Hydrometallurgy.

[18] Tao W., Zhu C., Xu Q., Li S., Xiong X., Cheng H., Zou X., Lu X. (2020). Electronic
Structure and Oxidation Mechanism of Nickel–Copper
Converter Matte from First-Principles Calculations. ACS Omega.

[19] Han S.-K., Gu C., Gong M., Yu S.-H. (2015). A Trialkylphosphine-Driven Chemical
Transformation Route to Ag- and Bi-Based Chalcogenides. J. Am. Chem. Soc..

[20] Sines I. T., Schaak R. E. (2011). Phase-Selective
Chemical Extraction of Selenium and
Sulfur from Nanoscale Metal Chalcogenides: A General Strategy for
Synthesis, Purification, and Phase Targeting. J. Am. Chem. Soc..

[21] Steimle B. C., Lord R. W., Schaak R. E. (2020). Phosphine-Induced
Phase Transition
in Copper Sulfide Nanoparticles Prior to Initiation of a Cation Exchange
Reaction. J. Am. Chem. Soc..

[22] Hoshino H., Yotsuyanagi T. (1985). Solubility
and Synergistic Extraction Equilibria for
[4-(2-Pyridylazo)­resorcinolato] Nickel­(II) Complexes. Bulletin of the Chemical Society of Japan.

[23] Thompson D., Hoffman A. S., Mansley Z. R., York S., Wang F., Zhu Y., Bare S. R., Chen J. (2024). Synthesis of Amorphous and Various
Phase-Pure Nanoparticles of Nickel Phosphide with Uniform Sizes via
a Trioctylphosphine-Mediated Pathway. Inorg.
Chem..

