# Supplementary material for: Concurrent Etching and Exchange Reactions During the Partial Replacement of Cu+ with Ni2+ in Copper Sulfide Nanorods
Source: Inorg Chem. 2026 Sep 9;65(37):21436–40. doi: 10.1021/acs.inorgchem.6c03606 (PMC13599380; doi:10.1021/acs.inorgchem.6c03606)
Supplement: Supplementary file 1 [file ic6c03606_si_001.pdf]

SUPPORTING INFORMATION

Concurrent Etching and Exchange Reactions During the Partial Replacement of Cu^{+} with Ni^{2+} in Copper Sulfide Nanorods

Chul-Hyun Jeong[1] and Raymond E. Schaak[1,2,3,*]

[1] *Department of Chemistry, The Pennsylvania State University, University Park, PA 16802, United States.*

[2] *Department of Chemical Engineering, The Pennsylvania State University, University Park, PA 16802, United States.*

[3] *Materials Research Institute, The Pennsylvania State University, University Park, PA 16802, Unites States.*

E-mail: res20@psu.edu

Table of Contents **Pages**

Experimental Details S2 – S5

Supplemental Tables (Tables S1 – S4) S6 – S9

Supplemental Figures (Figures S1 – S15) S10 – S24

Experimental Details

All syntheses and cation solution preparations were performed using a standard Schlenk line with a 50 mL three-neck round-bottom flask equipped with a heating mantle, gas flow adapter, reflux condenser, thermometer adapter, glass-encapsulated thermocouple, rubber septum, and magnetic stir bar.[1,2] Standard Schlenk line techniques were utilized to maintain air-free conditions. Reaction temperatures were controlled using a J-KEM Scientific temperature controller, unless otherwise noted.

Chemicals

Copper(II) nitrate trihydrate [$Cu(NO_3)_2{\cdot}3H_2O$, puriss. p.a., 99–104%], trioctylphosphine oxide [TOPO, 99%], 1-octadecene [ODE, technical grade, 90%], oleylamine [technical grade, 70%], tert-dodecanethiol [t-DDT, mixture of isomers, 98.5%], 1-dodecanethiol [1-DDT, ≥98%], nickel(II) chloride [$NiCl_2$, 98%], and cobalt(II) chloride [$CoCl_2$, 97%] were purchased from Sigma Aldrich. Nickel(II) acetate tetrahydrate [$Ni(CH_3COO)_2{\cdot}4H_2O$, $Ni(OAc)_2$, 99+%] was purchased from Alfa Aesar. Benzyl ether [99%] was purchased from Acros Organics. Trioctylphosphine [TOP, >85%] was purchased from TCI America. All solvents (hexanes, toluene, acetone, and isopropyl alcohol [IPA]) were of analytical grade. All chemicals were used as received without further purification.

Synthesis of roxbyite ($Cu_{1.8}S$) nanorods

Roxbyite ($Cu_{1.8}S$) nanorods were prepared following a modified literature procedure.[3] In this synthesis, a 50 mL three-neck round-bottom flask was equipped with a standard Schlenk line setup, and TOPO (2.9 g), $Cu(NO_3)_2{\cdot}3H_2O$ (281 mg), ODE (15 mL), and oleylamine (250 μL) were added to the flask in the order listed. Under vacuum, the mixture was heated to 60 °C with stirring for 30 min for degassing, during which the solution was clear bright blue. Separately, a 10:1 (v/v) t-DDT:1-DDT mixture (7.5 mL) was prepared in a septum-capped 20 mL vial and subjected to three vacuum-Ar cycles. After degassing, the flask was also subjected to three vacuum-Ar cycles and maintained under an outward Ar flow by connecting a tube hose to a bubbler through the rubber septum with a syringe needle. While maintaining this argon flow, the mixture was allowed to cool to approximately 50 °C by removing the first heating mantle. Meanwhile, a second heating mantle was preheated at maximum Variac output for 15 min. The second preheated mantle was then applied to the flask to raise the temperature rapidly. As the temperature increased and reached 75 °C over approximately 1.5 min, 7.5 mL of the 10:1 t-DDT:1-DDT mixture was rapidly injected using an Ar-purged syringe, resulting in a color change to clear yellow. For optimal nanorod quality, the temperature increased to 180 °C over approximately 3 min (within 5 min) after injection of the thiol mixture. Once the reaction mixture reached 180 °C, this temperature was maintained at 180 ± 5 °C for 25 min to allow complete growth of the $Cu_{1.8}S$ nanorods to the desired dimensions. During the first 3 min at 180 °C, the reaction mixture gradually darkened from clear yellow to brown and finally to an opaque brown suspension. After 25 min, the flask was removed from the heating mantle and cooled to near room temperature in a water bath. The product was transferred to centrifuge tubes, and a 1:1 (v/v) IPA:acetone mixture was added as an antisolvent. The suspension containing the antisolvent was centrifuged at 14,500 rpm (18,335 × *g*) for 2 min. After decanting the supernatant, the precipitate was re-dispersed in toluene (with brief sonication as needed), subject to an additional wash with the 1:1 IPA:acetone mixture, and collected again by centrifugation. The final product was re-dispersed in hexanes and stored in a 20 mL vial at ambient conditions for subsequent characterization and experiments. The as-synthesized $Cu_{1.8}S$ nanorods exhibited dimensions of 54 ± 3 × 22 ± 1 nm (n = 292) (**Figure S1**).

Preparation of cation stock solutions.

Cation stock solutions were prepared using a modified literature method.[3] The weighed amounts of metal precursors and the corresponding solvent compositions, targeting a concentration of 0.015 M, are listed in **Table S2**. Appropriate amounts of solvents and the metal precursors were added to a 50 mL three-neck round-bottom flask under a standard Schlenk line setup. TOPO is present as a solid at room temperature; therefore, it was fully melted by heating prior to use and delivered in liquid form for TOPO-containing solvent compositions. The flask was placed under vacuum and heated to 100 °C under stirring, and this temperature was maintained for 30 min to degas the solution. Following three vacuum-Ar cycles, the flask was kept under a blanket of Ar. The temperature was then raised to 160 °C and held for 60 min. During this process, solvent mixtures containing either oleylamine or TOPO completely dissolved the metal precursors, whereas solvent mixtures without both oleylamine and TOPO did not dissolve the metal precursors, leaving cloudy suspensions in the solvents. Next, the heating mantle was withdrawn, and the flask was allowed to cool to approximately 55 °C. The resulting solution was then transferred to a 40 mL septum-capped vial and stored under ambient conditions for use in subsequent experiments. Solidified cation stock solutions at room temperature were fully re-melted by heating, and cation stock solutions containing undissolved metal precursors were sonicated to ensure homogeneous dispersion prior to use.

Calculation for the volume of cation stock solution

Throughout this work, partial cation exchange reactions were conducted by controlling the volume of the incoming cation solution. First, the mass of the $Cu_{1.8}S$ nanorods was determined in order to calculate the total number of moles of $Cu_{1.8}S$ present. This was carried out by dispersing the nanorods (stored in hexanes) through sonication and transferring the resulting suspension into a pre-weighed 20 mL septum-capped vial. The hexanes were then evaporated under vacuum using a Schlenk line system until only dry nanorods remained. The mass of $Cu_{1.8}S$ nanorods was obtained by subtracting the mass of the empty vial from the final mass. The mass of surface-bound ligands was assumed to be negligible. Second, a target exchange fraction was defined to represent the proportion of Cu^+ in $Cu_{1.8}S$ to be replaced by the incoming cations. The required volume of the incoming cation solution was calculated using the following equation:

$$V_{cation\ solution} = \frac{(mmol_{Cu_{1.8}S}) \times (\frac{1.8\ mmol_{Cu^+}}{1\ mmol_{Cu_{1.8}S}})}{(ox.\ state\ of\ incoming\ cation) \times M_{cation\ solution}} \times fraction\ of\ exchange$$

In this equation, $V_{cation\ solution}$ represents the required volume of the cation solution for partial exchange. The *oxidation (ox.) state of incoming cation* is 2, since only divalent cations were employed. $M_{cation\ solution}$ denotes the molar concentration of the cation solution, and *fraction of exchange* indicates the proportion of Cu^+ targeted for replacement. An exchange fraction of 1 corresponds to complete stoichiometric substitution of Cu^+, whereas values below 1 imply partial exchange. For example, an exchange fraction of 0.33 signifies that a controlled volume of incoming cation solution was added to replace 1/3 of the Cu^+ cations in the $Cu_{1.8}S$ nanorods. A representative calculation for achieving 1/3 exchange using 10 mg of $Cu_{1.8}S$ nanorods is provided below.

$$\frac{(0.068\ \mathrm{mmol_{Cu_{1.8}S}}) \times (\frac{1.8\ \mathrm{mmol_{Cu^+}}}{1\ \mathrm{mmol_{Cu_{1.8}S}}})}{(2) \times 0.015\ \mathrm{M_{cation\ solution}}} \times 0.33 = 1.35\ \mathrm{mL\ of\ cation\ solution}$$

Partial cation exchange reactions

Partial cation exchange reactions were performed on roxbyite $Cu_{1.8}S$ nanorods following a protocol adapted from published procedures with minor modifications.[3] In each exchange reaction, a 50 mL three-neck round-bottom flask was equipped with a standard Schlenk line setup. The solvent compositions listed in **Table S3** were added to the flask, along with the appropriate volume of the corresponding cation solution required to achieve the targeted exchange fraction (**Table S3**). The solution mixture was heated to 100 °C under vacuum with stirring and degassed for 30 min. The flask was then cycled three times between vacuum and Ar, and kept under a blanket of Ar. Then, the temperature was raised to 140 °C and held at this temperature to be ready for the exchange reaction. Separately, TOP (3 mL) was added to a 20 mL septum-capped vial containing dried $Cu_{1.8}S$ nanorods (10 mg). After addition of TOP, the vial was immediately placed under vacuum through the Schlenk line setup to minimize exposure of TOP to air, followed by three vacuum-Ar cycles. The vial was then kept under Ar and sonicated for 45 min to ensure complete dispersion of the $Cu_{1.8}S$ nanorods in TOP. After 45 min sonication, the resulting $Cu_{1.8}S$/TOP suspension was rapidly injected into the reaction flask maintained at 140 °C, and the exchange reaction was allowed to proceed for 90 min. Aliquots were withdrawn at 3, 17, and 50 min, if needed. After 90 min, the reaction flask was removed from the heating mantle and cooled in an ice bath to approximately 10 °C. The cooled mixture was transferred to centrifuge tubes containing a pre-cooled 1:1 (v/v) mixture of IPA:acetone and centrifuged at 14,500 rpm (18,335 × g) for 2 min. The supernatant was discarded, and the precipitate was redispersed in toluene with brief sonication. The particles were washed two additional times using the same 1:1 IPA:acetone antisolvent, followed by centrifugation and re-dispersion in toluene. During the final re-dispersion step, one drop of oleylamine was added to improve surface ligand coverage and enhance particle dispersibility for STEM analysis. The final products were dispersed in hexanes and stored under ambient conditions for further characterization.

Full cation exchange reactions

Full exchange reactions with either Ni^{2+} or Co^{2+} were conducted on roxbyite $Cu_{1.8}S$ nanorods for XRD analysis, modifying a published procedure.[3] In each exchange reaction, a 50 mL three-neck round-bottom flask was equipped with a standard Schlenk line setup. Benzyl ether (5 mL), oleylamine (2 mL), and ODE (1 mL) were added to the flask, along with 8.2 mL of either standard $NiCl_2$ solution or standard $CoCl_2$ solution listed in **Table S2** to achieve complete exchange. The solution mixture was heated to 100 °C under vacuum with stirring and degassed for 30 min. The flask was then cycled three times between vacuum and Ar and kept under a blanket of Ar. The temperature was then elevated to 140 °C and maintained at this setpoint to be ready for the exchange reaction. Separately, TOP (3 mL) was added to a 20 mL septum-capped vial containing dried $Cu_{1.8}S$ nanorods (20 mg). After addition of TOP, the vial was immediately placed under vacuum through the Schlenk line setup to minimize exposure of TOP to air, followed by three vacuum-Ar cycles. The vial was then kept under Ar and sonicated for 45 min to ensure complete dispersion of the $Cu_{1.8}S$ nanorods in TOP. The resulting $Cu_{1.8}S$/TOP suspension was rapidly injected into the reaction flask held at 140 °C, and the exchange reaction proceeded for 90 min. For exchange reactions conducted at 100 °C, the reaction was allowed to proceed for 60 min. After 60 or 90 min, the reaction flask was removed from the heating mantle and cooled in an ice bath to approximately 10 °C. The cooled reaction mixture was transferred to centrifuge tubes containing a pre-cooled 1:1 (v/v) IPA:acetone and centrifuged at 14,500 rpm (18,335 × g) for 2 min. The supernatant was discarded, and the precipitate was re-dispersed in toluene with brief sonication. The particles were washed two additional times using the same 1:1 IPA:acetone antisolvent, followed by centrifugation and re-dispersion in toluene. Before the final re-dispersion step, one drop of oleylamine was added to the toluene to recover surface ligand passivation and improve particle dispersibility for STEM analysis. The final products were dispersed in hexanes and stored under ambient conditions for further characterization.

Characterization

Powder X-ray diffraction (XRD) patterns were collected using a Malvern Panalytical Empyrean diffractometer equipped with a Cu Kα radiation source. Samples for XRD analysis were prepared by drop-casting onto zero-background silicon holders. Reference crystal structures were generated from reported structural data using CrystalMaker (CrystalMaker Software Ltd., Oxford, UK) or obtained as crystallographic information files from the Inorganic Crystal Structure Database (ICSD). Reference diffraction patterns were simulated, and the experimental XRD patterns were analyzed, using CrystalDiffract (CrystalMaker Software Ltd., Oxford, UK). High-angle annular dark-field scanning transmission electron microscopy (HAADF-STEM) and STEM energy-dispersive X-ray spectroscopy (STEM-EDS) measurements were performed using a Thermo Scientific Talos F200X S/TEM operated at 200 kV and equipped with a SuperX EDS detector. Samples for electron microscopy were prepared by drop-casting dilute nanoparticle dispersions onto Au TEM grids. STEM-EDS elemental maps were processed and analyzed using Thermo Scientific Velox software. Elemental distributions and compositions were evaluated using the Cu Kα, Co Kα, Ni Kα, and S Kα X-ray emission lines. Particle dimensions and heterostructure domain sizes were measured from HAADF-STEM images and STEM-EDS element maps using ImageJ software.

Supplemental Tables

Table S1. Average length × width dimensions of copper sulfide and heterometal sulfide regions in nanorods. Values are reported as mean ± standard deviation; n indicates the number of measured particles or domains. These values were used to generate the plots in **Figure 1**.

Reactions	$Cu_{1.8}S$ nanorods	Partial Ni^{2+} reaction	Partial Ni^{2+}/Co^{2+} reaction	Partial Co^{2+} reaction
Copper sulfide sizes (nm)	54 ± 3 × 22 ± 1 (n = 292)	12 ± 5 × 16 ± 3 (n = 41)	20 ± 8 × 13 ± 3 (n = 48)	34 ± 6 × 20 ± 1 (n = 69)
Heterometal sulfide sizes (nm)	N/A	Nickel Sulfide 28 ± 6 × 12 ± 2 (n = 87)	Nickel-Cobalt Sulfide 17 ± 4 × 16 ± 3 (n = 54)	Cobalt Sulfide 15 ± 4 × 17 ± 1 (n = 63)

Table S2. Regional STEM-EDS atomic fraction analysis of nickel sulfide and copper sulfide regions in aliquots taken during partial Ni^{2+} exchange reaction. Values are reported as mean ± standard deviation from three STEM-EDS elemental maps for each sample.

3 min aliquot	Nickel Sulfide Region	Ni fraction (%)	**47.6 ± 0.6**
		S fraction (%)	52.4 ± 0.6
	Copper Sulfide Region	Cu fraction (%)	60.9 ± 0.9
		S fraction (%)	39.1 ± 0.9
17 min aliquot	Nickel Sulfide Region	Ni fraction (%)	**49.6 ± 0.4**
		S fraction (%)	50.4 ± 0.4
	Copper Sulfide Region	Cu fraction (%)	60 ± 1
		S fraction (%)	40 ± 1
50 min aliquot	Nickel Sulfide Region	Ni fraction (%)	**50.2 ± 0.7**
		S fraction (%)	49.8 ± 0.7
	Copper Sulfide Region	Cu fraction (%)	61.1 ± 0.4
		S fraction (%)	38.9 ± 0.4
90 min aliquot	Nickel Sulfide Region	Ni fraction (%)	**55 ± 1**
		S fraction (%)	45 ± 1
	Copper Sulfide Region	Cu fraction (%)	59 ± 1
		S fraction (%)	41 ± 1

Table S3. Cation stock solutions used for partial/full exchange reactions. The table summarizes the metal precursor, precursor mass, solvent composition, and observed solubility for each stock solution prepared with oleylamine, TOPO, or neither ligand.

Cation stock solution	Metal precursor	Precursor mass	Solvent composition	Solubility in stock solution
Standard $NiCl_2$ solution	$NiCl_2$	49 mg	Benzyl ether (15 mL), oleylamine (8 mL), ODE (2 mL)	Fully dissolved
Standard $CoCl_2$ solution	$CoCl_2$	49 mg	Benzyl ether (15 mL), oleylamine (8 mL), ODE (2 mL)	Fully dissolved
$NiCl_2$ solution (TOPO, no oleylamine)	$NiCl_2$	49 mg	Benzyl ether (15 mL), TOPO (8 mL), ODE (2 mL)	Fully dissolved
$NiCl_2$ solution (no oleylamine, no TOPO)	$NiCl_2$	49 mg	Benzyl ether (20 mL), ODE (5 mL)	Undissolved
$Ni(OAc)_2$ solution (oleylamine)	$Ni(OAc)_2 \cdot 4H_2O$	94 mg	Benzyl ether (15 mL), oleylamine (8 mL), ODE (2 mL)	Fully dissolved
$Ni(OAc)_2$ solution (TOPO, no oleylamine)	$Ni(OAc)_2 \cdot 4H_2O$	94 mg	Benzyl ether (15 mL), TOPO (8 mL), ODE (2 mL)	Fully dissolved
$Ni(OAc)_2$ solution (no oleylamine, no TOPO)	$Ni(OAc)_2 \cdot 4H_2O$	94 mg	Benzyl ether (20 mL), ODE (5 mL)	Undissolved

Table S4. Summary of partial cation exchange reaction conditions, including solvent composition, cation stock solution, target exchange fraction, cation solution volume, and corresponding characterization figures.

Partial exchange reactions	Solvent composition	Cation stock solution (from **Table S2**)	Target exchange fraction	Cation solution volume	Results
Single Ni^{2+} exchange	Benzyl ether (5 mL), oleylamine (2 mL), ODE (1 mL)	Standard $NiCl_2$ solution	1/3	1.35 mL	Figure 1b, 2a, 3a, S2, S5
Single Co^{2+} exchange	Benzyl ether (5 mL), oleylamine (2 mL), ODE (1 mL)	Standard $CoCl_2$ solution	1/3	1.35 mL	Figure 1d, S4
Simultaneous Ni^{2+}/Co^{2+} exchange	Benzyl ether (5 mL), oleylamine (2 mL), ODE (1 mL)	Standard $NiCl_2$ + $CoCl_2$ solutions	1/6 each	0.69 mL / 0.69 mL	Figure 1c, 2b, S3, S6
Ni^{2+} exchange (TOPO no oleylamine)	Benzyl ether (5 mL), TOPO (2 mL), ODE (1 mL)	$NiCl_2$ solution (TOPO, no oleylamine)	1/3	1.35 mL	Figure 3c, S7
Ni^{2+} exchange (no oleylamine, no TOPO)	Benzyl ether (6 mL), ODE (2 mL)	$NiCl_2$ solution (no oleylamine, no TOPO)	1/3	1.35 mL	Figure 3e, S8
Ni^{2+} exchange (oleylamine, no Cl^-)	Benzyl ether (5 mL), OLAM (2 mL), ODE (1 mL)	$Ni(OAc)_2$ solution (oleylamine)	1/3	1.35 mL	Figure 3b, S9
Ni^{2+} exchange (TOPO, no oleylamine, no Cl^-)	Benzyl ether (5 mL), TOPO (2 mL), ODE (1 mL)	$Ni(OAc)_2$ solution (TOPO, no oleylamine)	1/3	1.35 mL	Figure 3d, S10
Ni^{2+} exchange (no oleylamine, no TOPO, no Cl^-)	Benzyl ether (6 mL), ODE (2 mL)	$Ni(OAc)_2$ solution (no oleylamine, no TOPO)	1/3	1.35 mL	Figure 3f, S11

Supplemental Figures

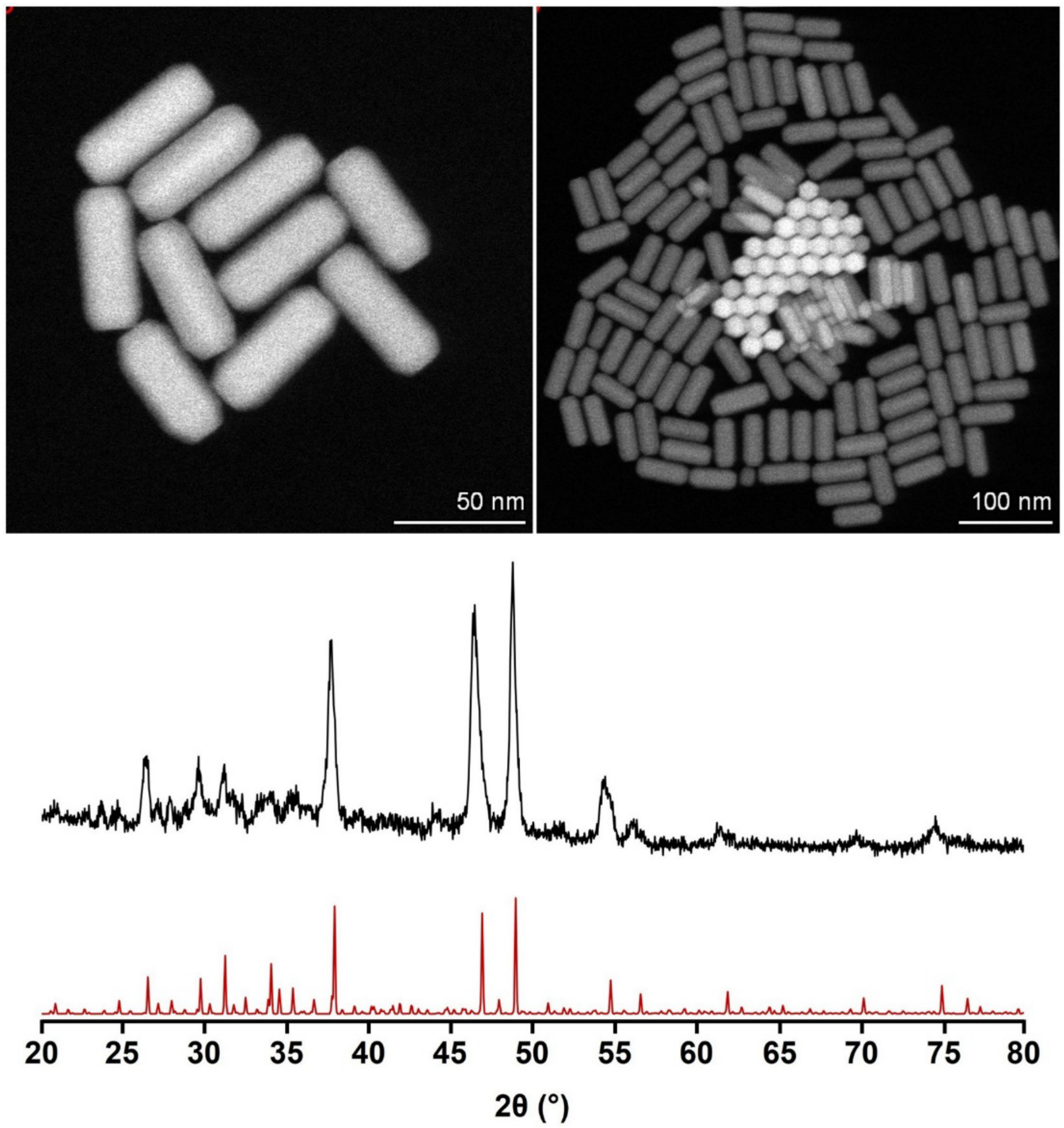

Figure S1. HAADF-STEM images and powder XRD characterization of $Cu_{1.8}S$ nanorods. Experimental XRD pattern of the $Cu_{1.8}S$ nanorods is shown in black and compared with the reference pattern for roxbyite $Cu_{1.8}S$,[4] shown in red. Size analysis of 292 nanoparticles gave average nanorod dimensions of 54 ± 3 nm (length) by 22 ± 1 nm (width). Particle size analysis from the HAADF-STEM images was carried out with ImageJ software.

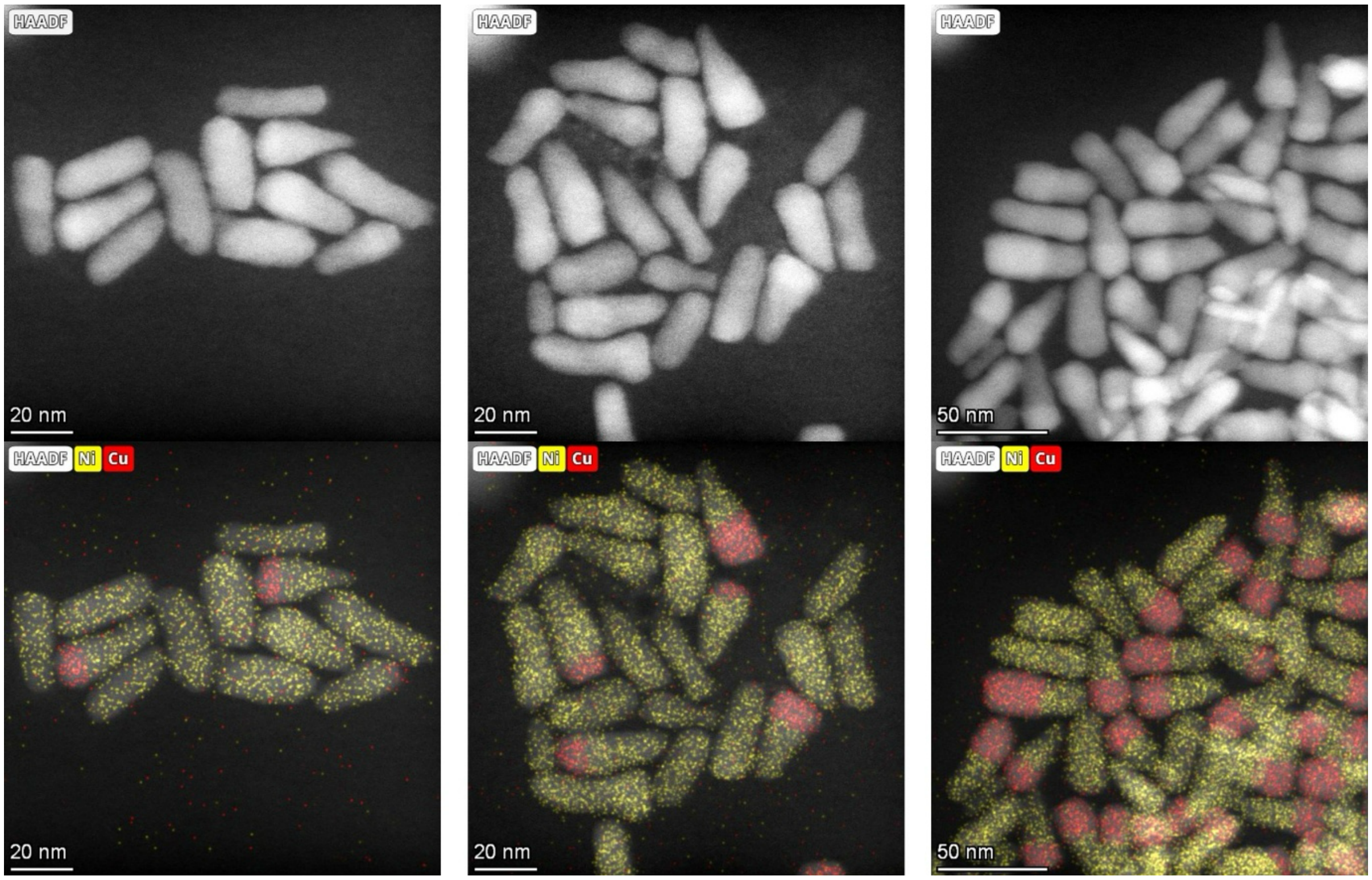

Figure S2. HAADF-STEM images and overlaid STEM-EDS element maps of products from a 90 min partial Ni^{2+} (1/3) exchange reaction. Emission lines used were Cu Kα (red) and Ni Kα (yellow).

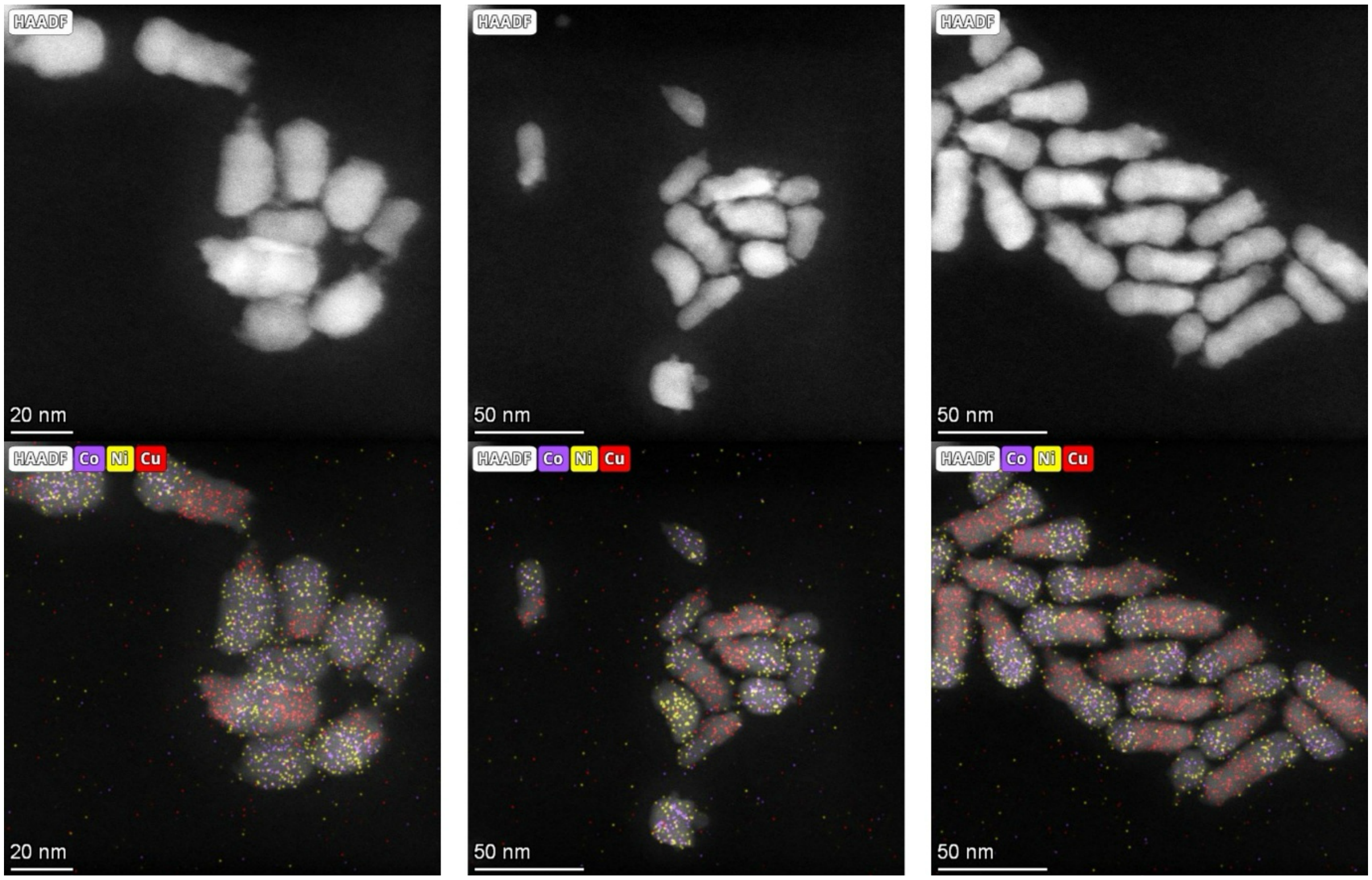

Figure S3. HAADF-STEM images and overlaid STEM-EDS element maps of products from a 90 min partial Ni^{2+}/Co^{2+} (1/6 each) exchange reaction. Emission lines used were Cu Kα (red), Co Kα (purple) and Ni Kα (yellow).

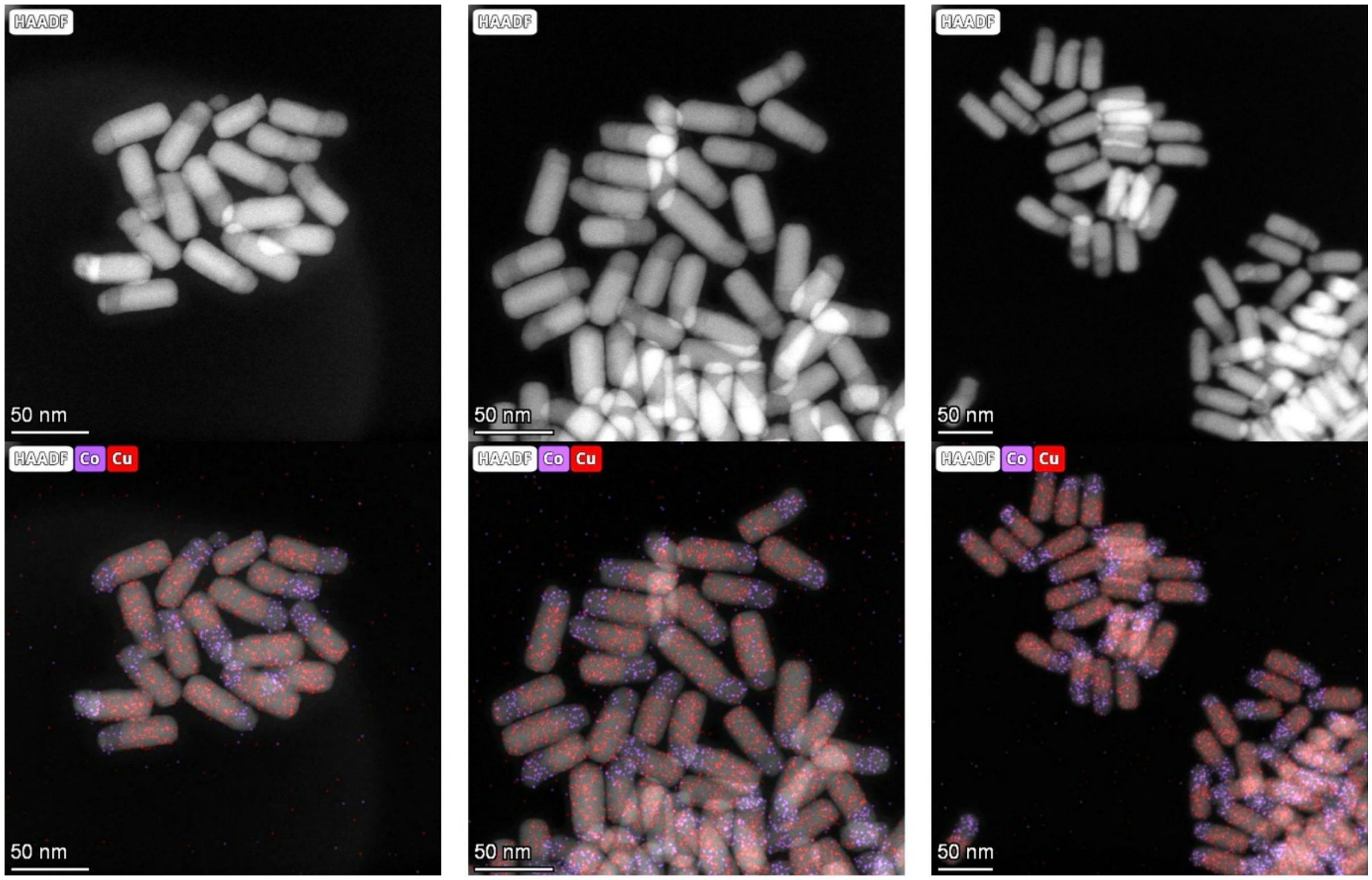

Figure S4. HAADF-STEM images and overlaid STEM-EDS element maps of products from a 90 min partial Co^{2+} (1/3) exchange reaction. Emission lines used were Cu Kα (red) and Co Kα (purple).

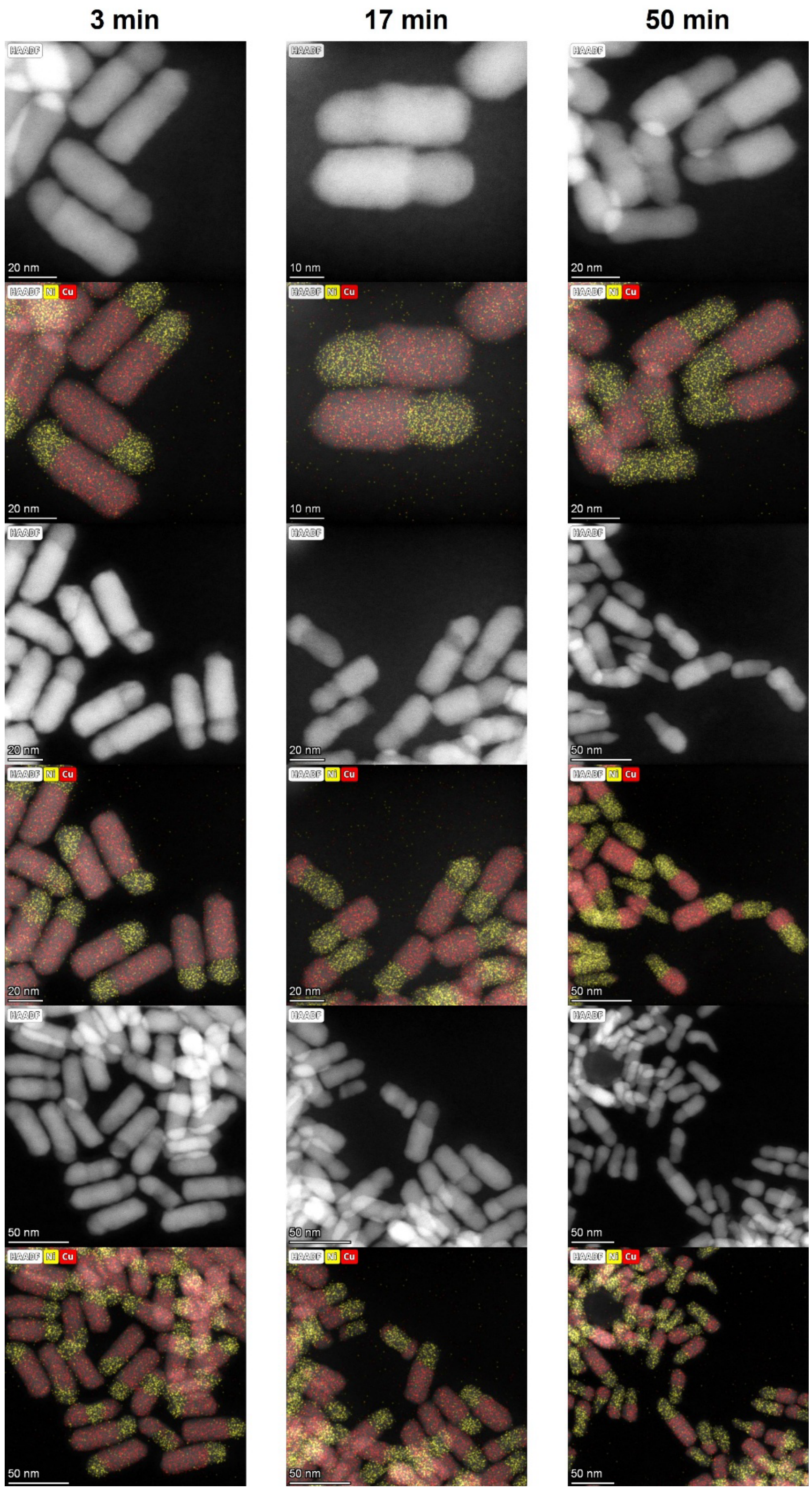

Figure S5. HAADF-STEM images and overlaid STEM-EDS element maps of aliquot products collected at 3, 17, and 50 min during a 90 min partial Ni^{2+} (1/3) exchange reaction. Emission lines used were Cu Kα (red) and Ni Kα (yellow).

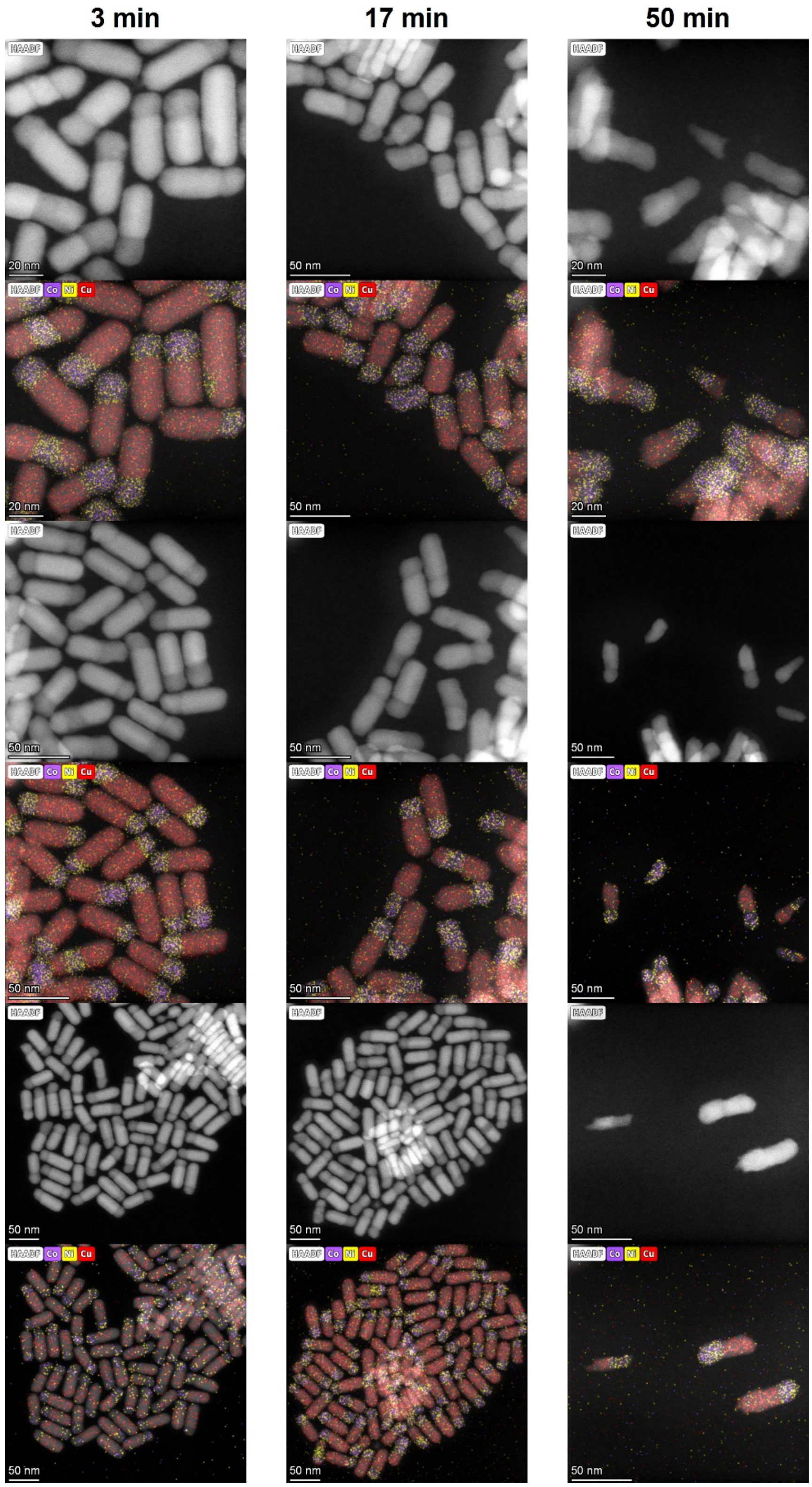

Figure S6. HAADF-STEM images and overlaid STEM-EDS element maps of aliquot products collected at 3, 17, and 50 min during a 90 min partial Ni^{2+}/Co^{2+} (1/6 each) exchange reaction. Emission lines used were Cu Kα (red), Co Kα (purple) and Ni Kα (yellow).

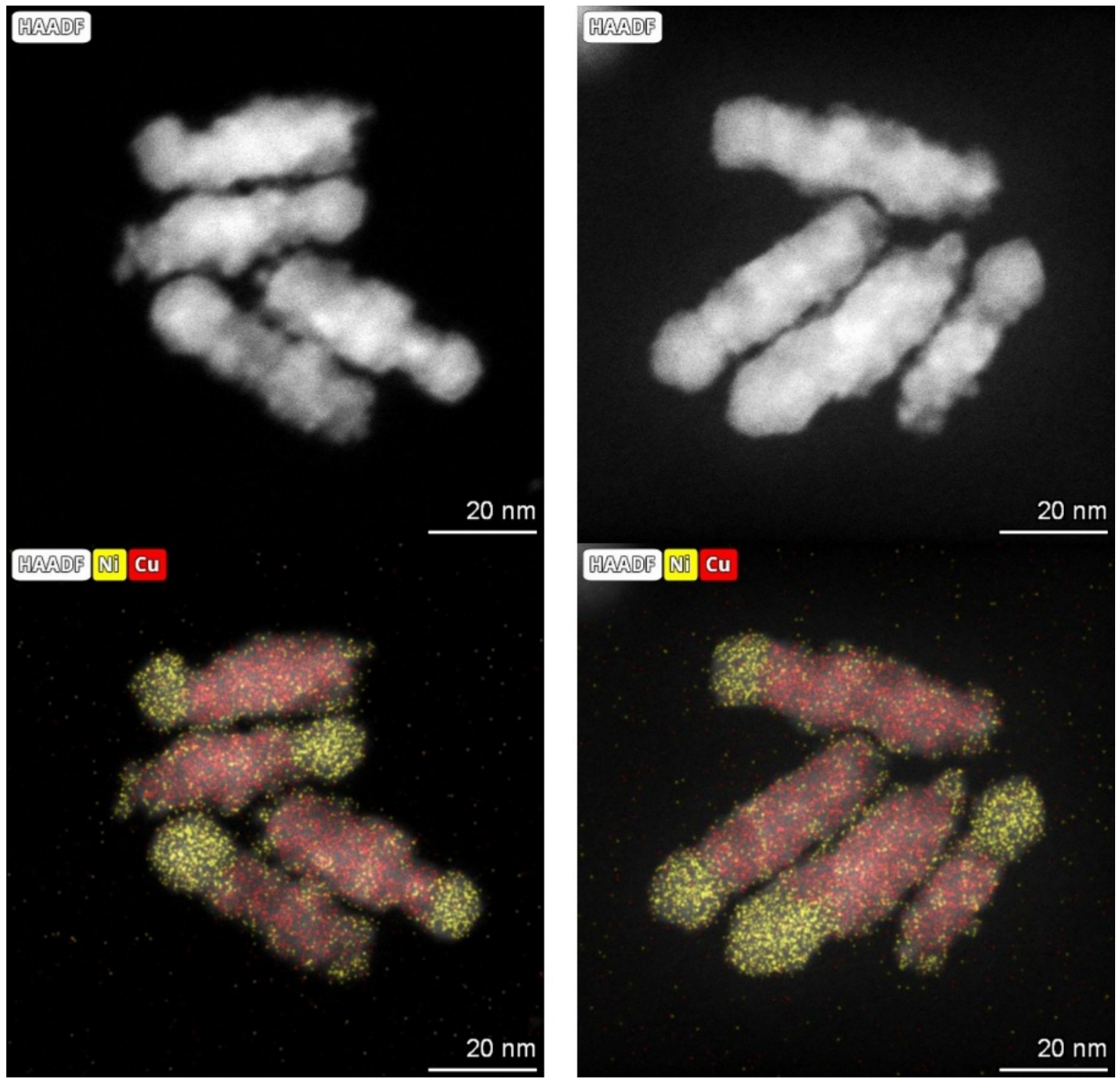

Figure S7. HAADF-STEM images and overlaid STEM-EDS element maps of products from a 90 min partial Ni^{2+} (1/3) exchange reaction ($NiCl_2$, without oleylamine, with TOPO). Emission lines used were Cu Kα (red) and Ni Kα (yellow).

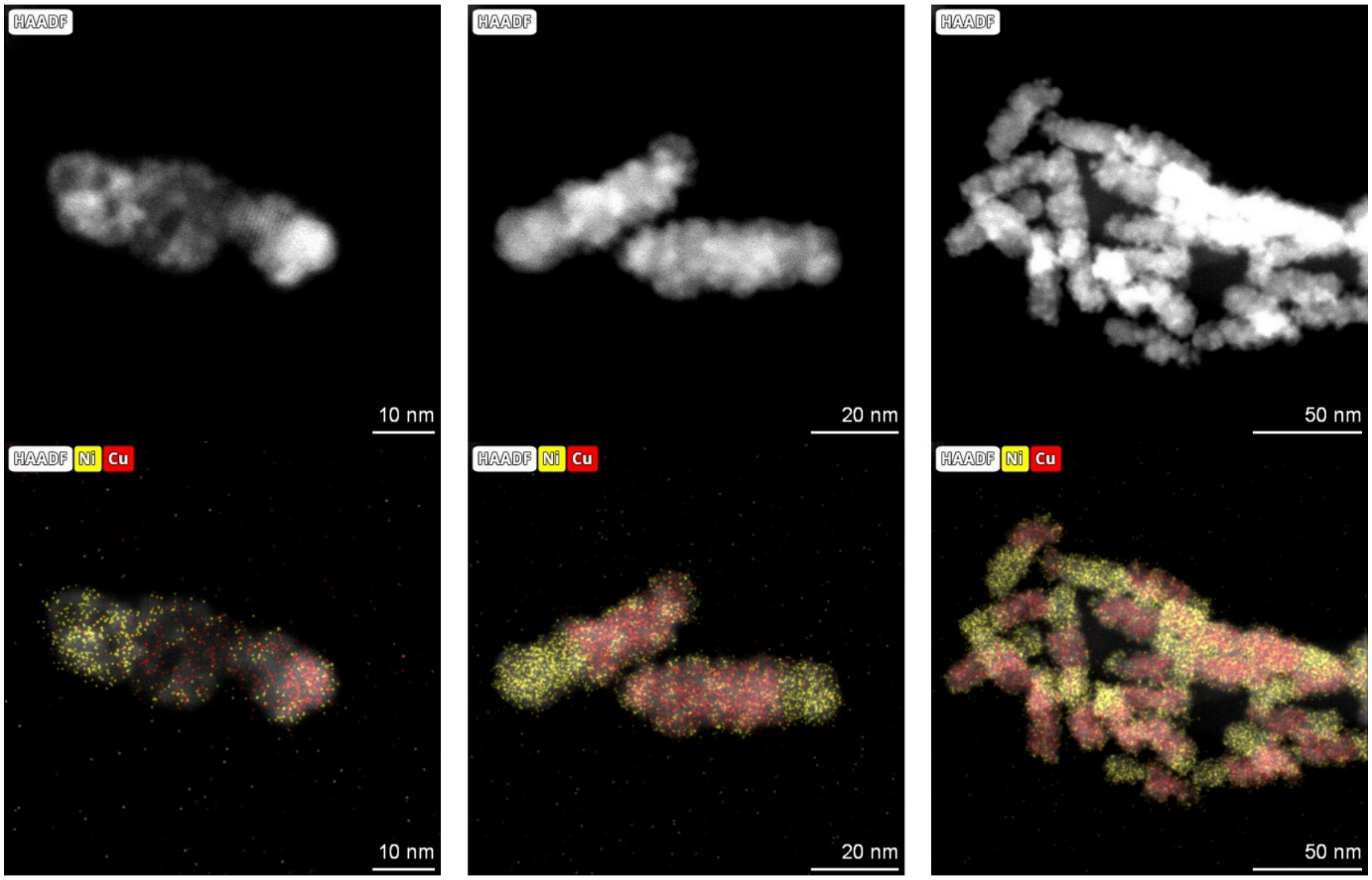

Figure S8. HAADF-STEM images and overlaid STEM-EDS element maps of products from a 90 min partial Ni^{2+} (1/3) exchange reaction ($NiCl_2$, without oleylamine, without TOPO). Emission lines used were Cu Kα (red) and Ni Kα (yellow).

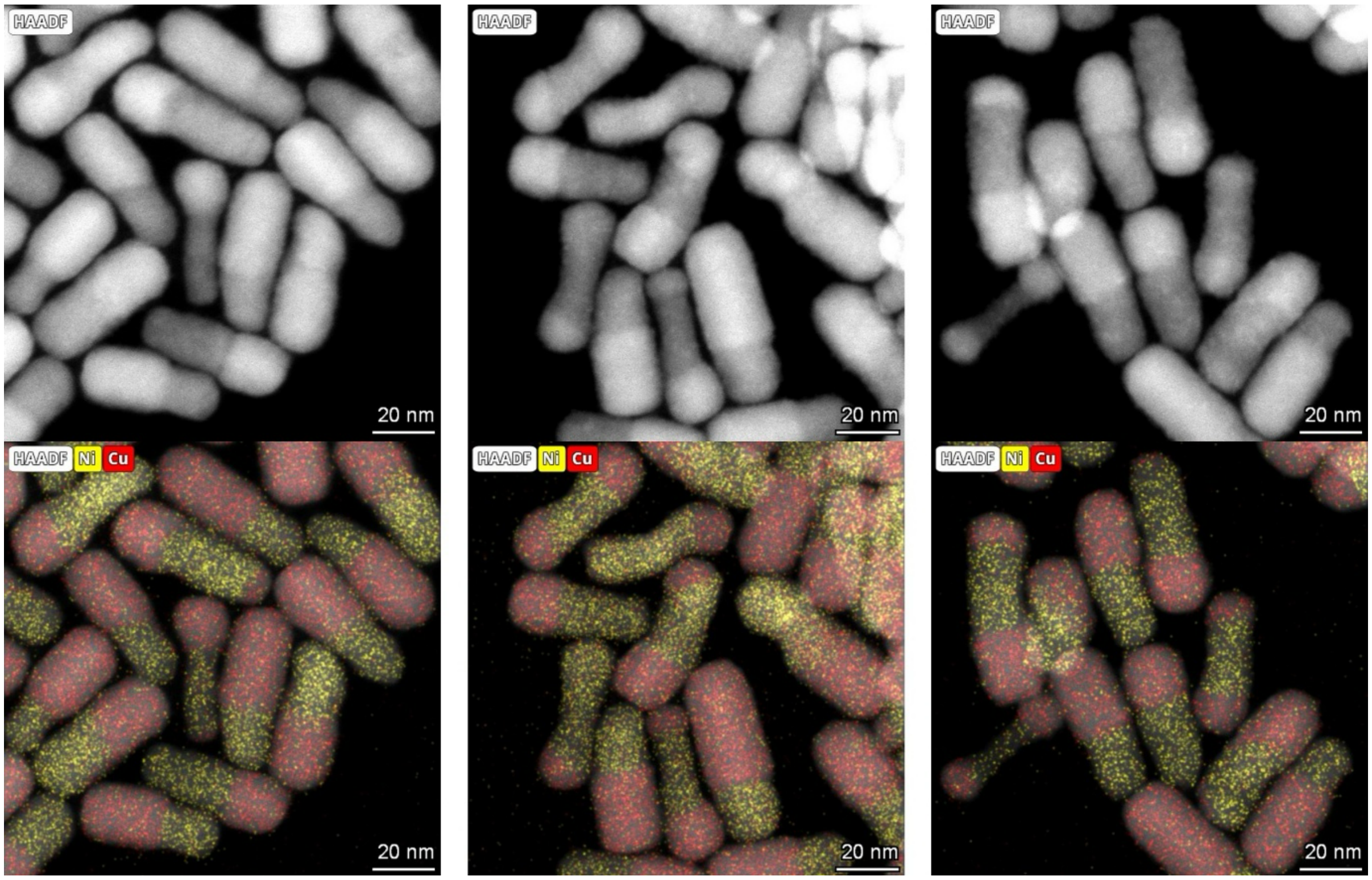

Figure S9. HAADF-STEM images and overlaid STEM-EDS element maps of products from a 90 min partial Ni^{2+} (1/3) exchange reaction ($Ni(OAc)_2$, with oleylamine, without TOPO). Emission lines used were Cu Kα (red) and Ni Kα (yellow).

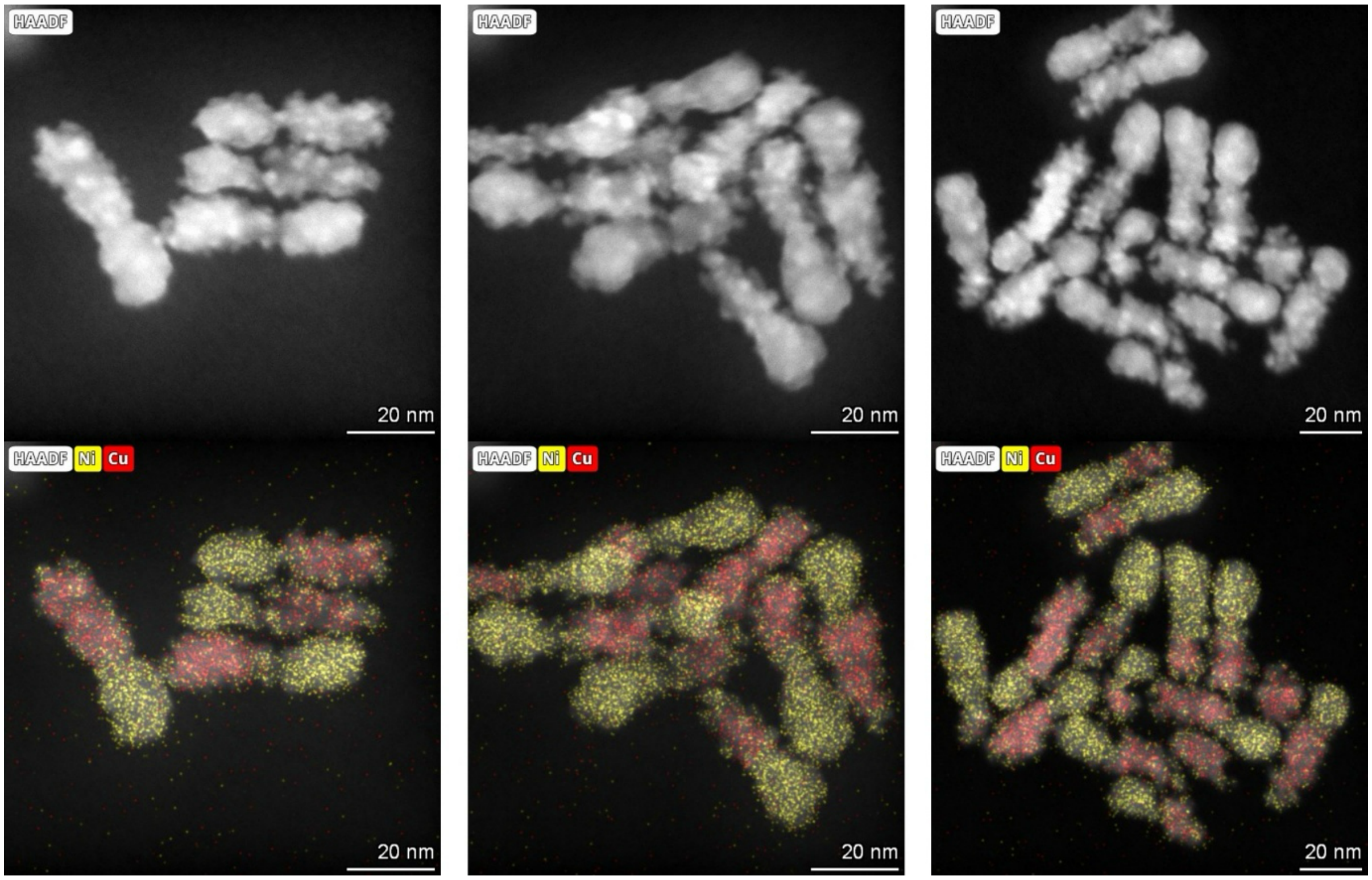

Figure S10. HAADF-STEM images and overlaid STEM-EDS element maps of products from a 90 min partial Ni^{2+} (1/3) exchange reaction ($Ni(OAc)_2$, without oleylamine, with TOPO). Emission lines used were Cu Kα (red) and Ni Kα (yellow).

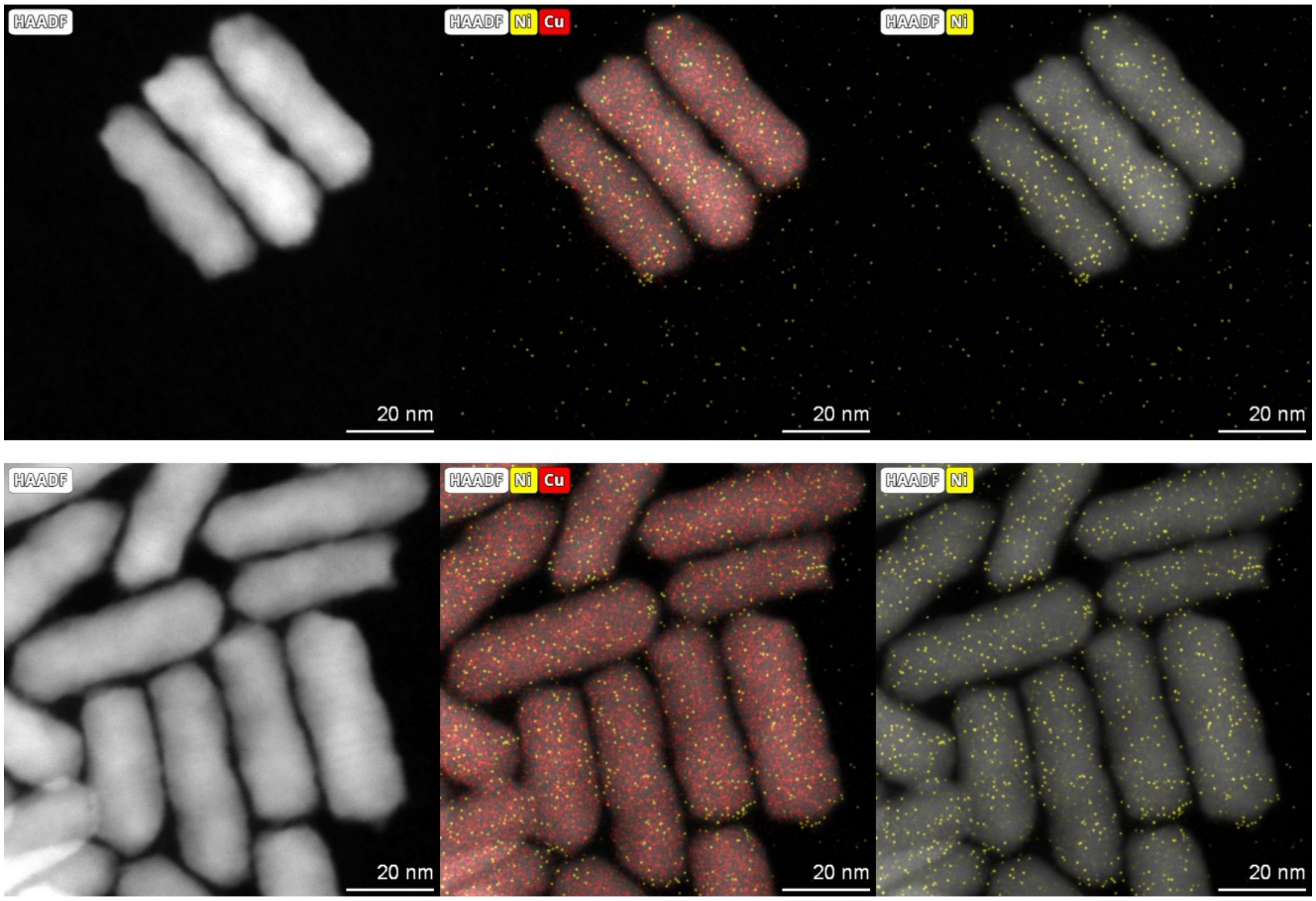

Figure S11. HAADF-STEM images and overlaid STEM-EDS element maps of products from a 90 min partial Ni^{2+} (1/3) exchange reaction ($Ni(OAc)_2$, without oleylamine, without TOPO). Emission lines used were Cu Kα (red) and Ni Kα (yellow).

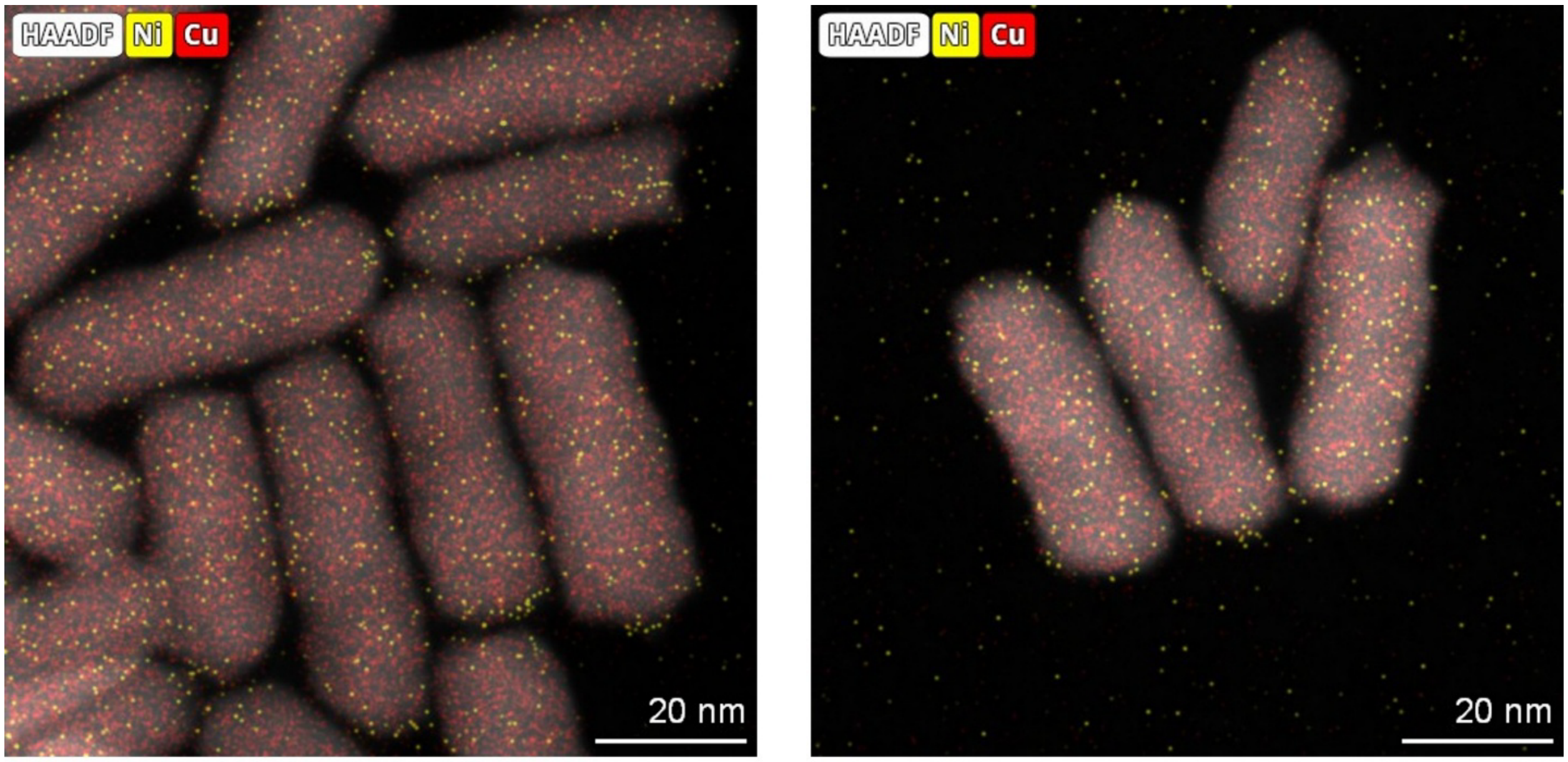

Z	Element	Family	Atomic Fraction (%)
28	Ni	K	1.73
29	Cu	K	98.27

Z	Element	Family	Atomic Fraction (%)
28	Ni	K	1.41
29	Cu	K	98.59

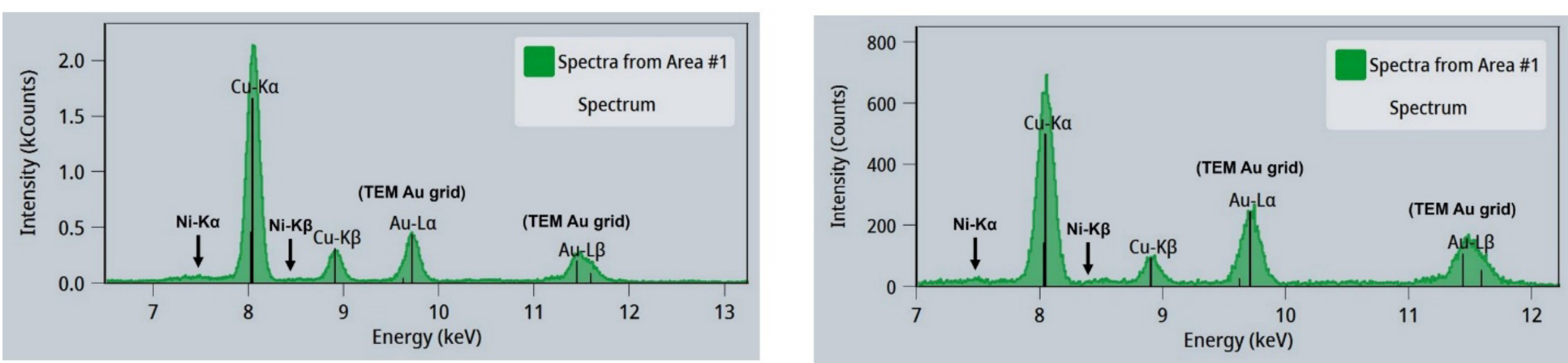

Figure S12. HAADF-STEM images, overlaid STEM-EDS element maps, STEM-EDS quantification data, and EDS spectra of products from a 90 min partial Ni^{2+} (1/3) exchange reaction ($Ni(OAc)_2$, without oleylamine, without TOPO). Emission lines used were Cu Kα (red) and Ni Kα (yellow).

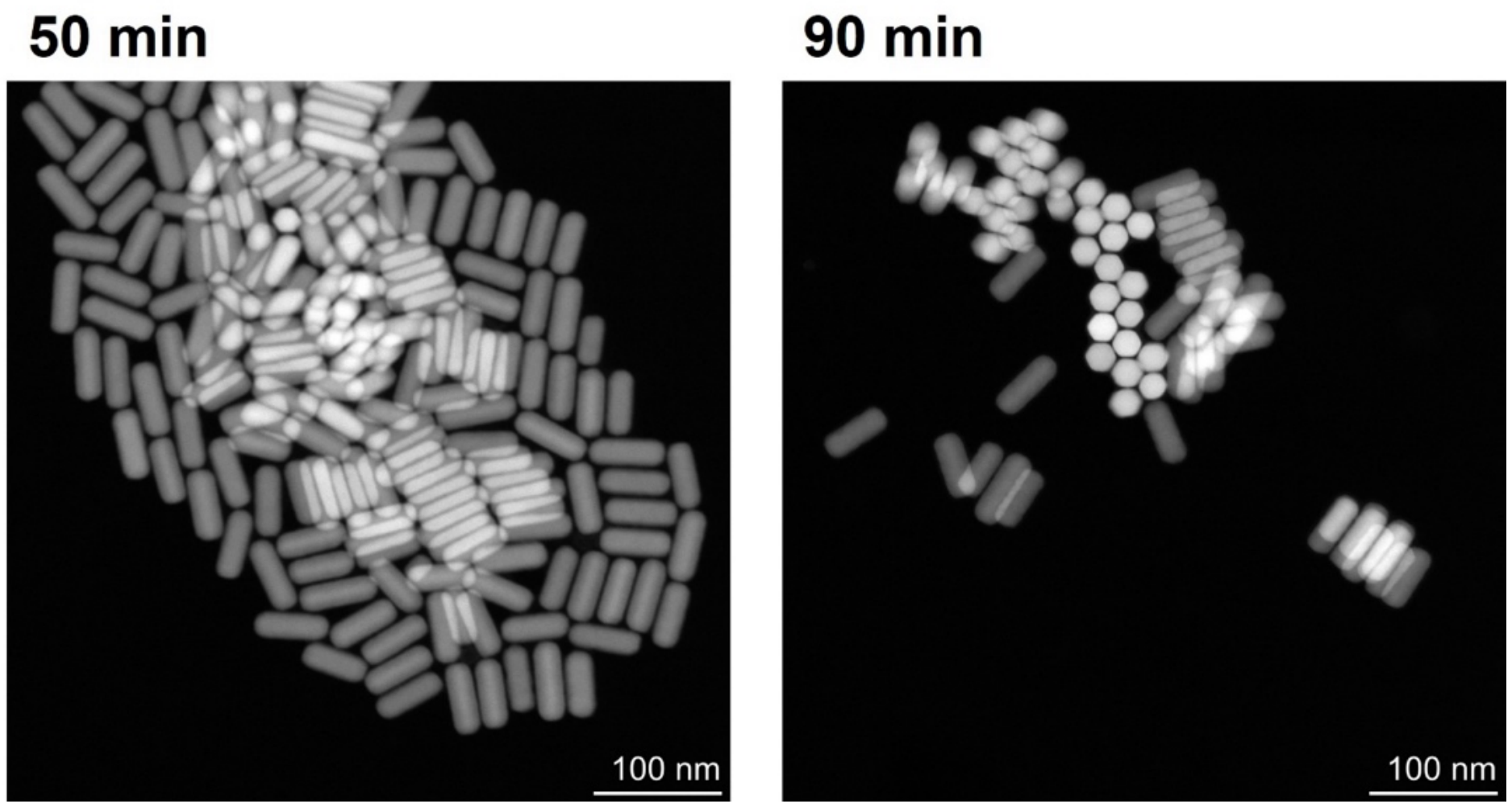

Figure S13. HAADF-STEM images of $Cu_{1.8}S$ nanorods after control experiments at 140 °C for 50 and 90 min in the presence of TOP and oleylamine but without added metal salts.

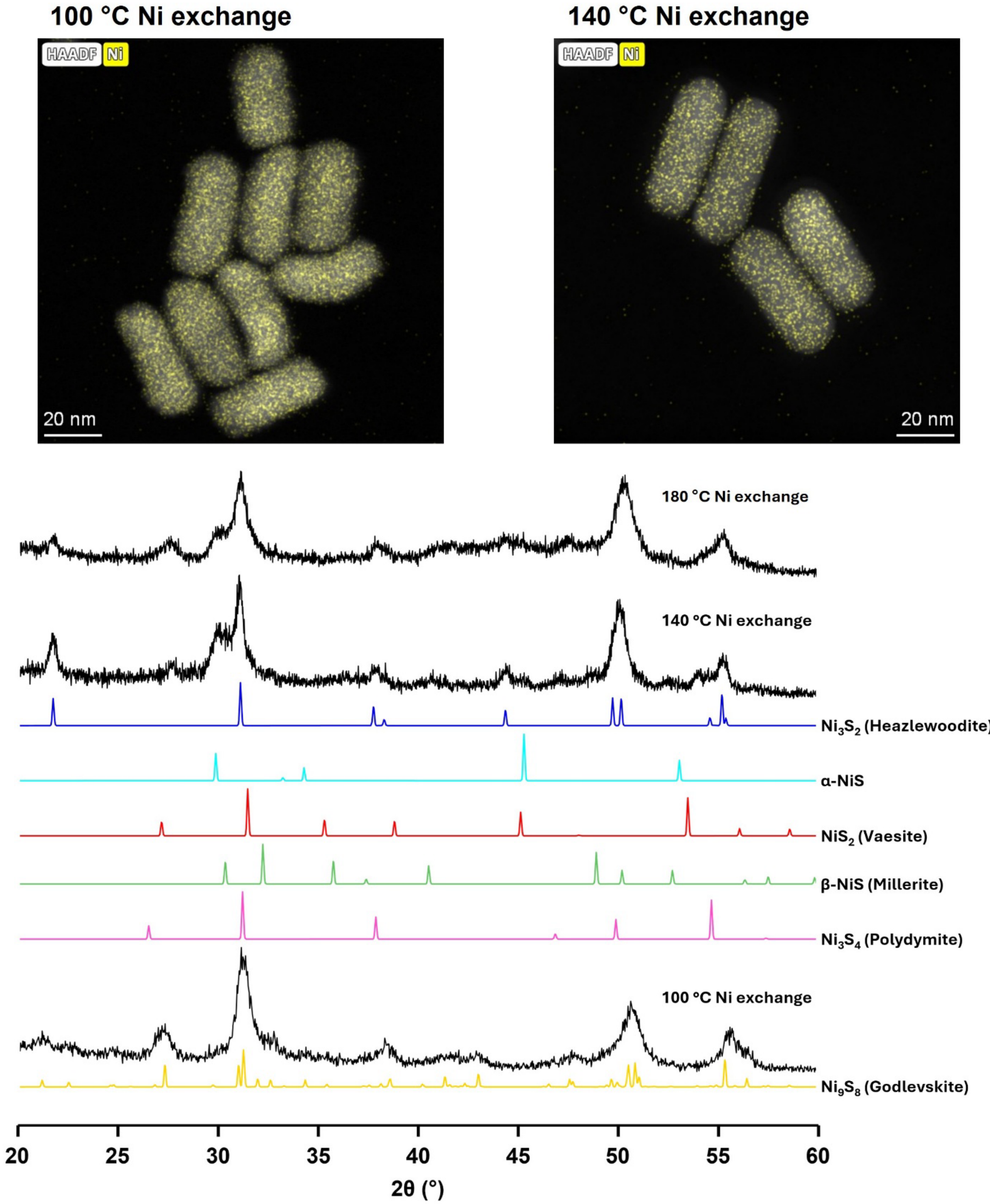

Figure S14. STEM-EDS element maps overlaid on HAADF-STEM images of products obtained from full Ni^{2+} exchange reactions at 100 and 140 °C. Emission line used was Ni Kα (yellow). Experimental XRD patterns are shown in black and compared with reference patterns for Ni_9S_8 (godlevskite),[5] Ni_3S_4 (polydymite),[6] β-NiS (millerite),[7] NiS_2 (Vaesite),[8] α-NiS,[9] and Ni_3S_2 (heazlewoodite: 36338 ICSD).

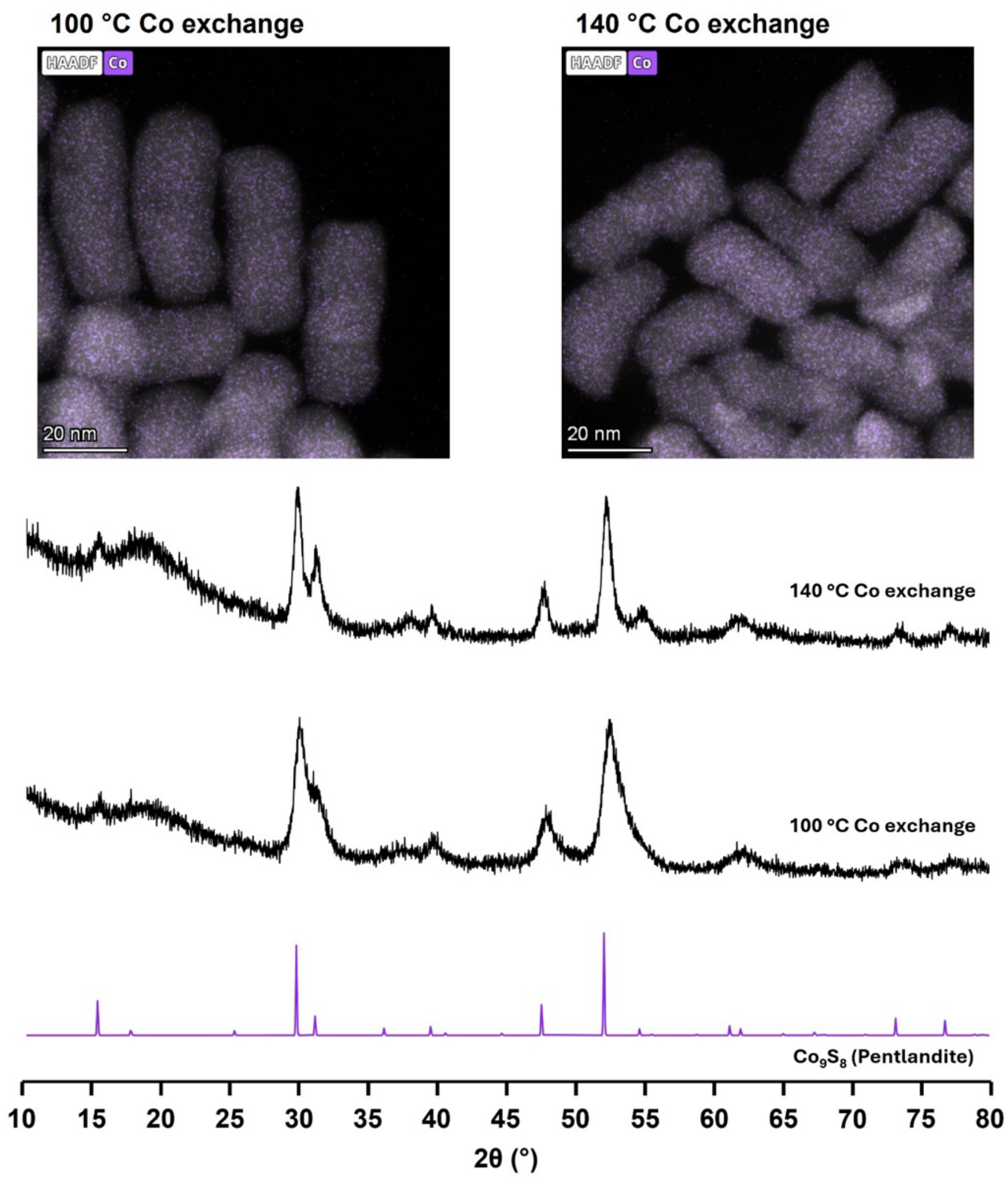

Figure S15. STEM-EDS element maps overlaid on HAADF-STEM images of products obtained from full Co^{2+} exchange reactions at 100 and 140 °C. Emission line used was Co Kα (purple). Experimental XRD patterns are shown in black and compared with reference patterns for Co_9S_8 (pentlandite).[10]

References

(1) Steimle, B. C.; Fagan, A. M.; Butterfield, A. G.; Lord, R. W.; McCormick, C. R.; Di Domizio, G. A.; Schaak, R. E. Experimental Insights into Partial Cation Exchange Reactions for Synthesizing Heterostructured Metal Sulfide Nanocrystals. *Chemistry of Materials* **2020**, *32* (13), 5461-5482.

(2) Borys, A. M. An Illustrated Guide to Schlenk Line Techniques. *Organometallics* **2023**, *42* (3), 182-196.

(3) Jeong, C.-H.; Schaak, R. E. Thermal Control of Material Placement in Heterostructured Metal Sulfide Nanorods. *Chemistry of Materials* **2026**, *38* (10), 5039-5048.

(4) Mumme, W. G.; Gable, R. W.; Petříček, V. THE CRYSTAL STRUCTURE OF ROXBYITE, $Cu_{58}S_{32}$. *The Canadian Mineralogist* **2012**, *50* (2), 423-430.

(5) Fleet, M. Structure of godlevskite, Ni_9S_8. *Acta Crystallographica Section C* **1987**, *43* (12), 2255-2257.

(6) Vaughan, D. J.; Craig, J. R. The crystal chemistry of iron-nickel thiospinels. *American Mineralogist* **1985**, *70* (9-10), 1036-1043.

(7) Grice, J. D.; Ferguson, R. B. Crystal structure refinement of Millerite (beta -NiS). *The Canadian Mineralogist* **1974**, *12* (4), 248-252.

(8) Nowack, E.; Schwarzenbach, D.; Hahn, T. Charge densities in CoS_2 and NiS_2 (pyrite structure). *Acta Crystallographica Section B* **1991**, *47* (5), 650-659.

(9) Sowa, H.; Ahsbahs, H.; Schmitz, W. X-ray diffraction studies of millerite NiS under non-ambient conditions. *Physics and Chemistry of Minerals* **2004**, *31* (5), 321-327.

(10) Geller, S. Refinement of the crystal structure of Co_9S_8. *Acta Crystallographica* **1962**, *15* (12), 1195-1198.
